# Supplementary material for: Unveiling Genomic Islands Hosting Antibiotic Resistance Genes and Virulence Genes in Foodborne Multidrug-Resistant Patho-Genic Proteus vulgaris
Source: Biology (Basel). 2025 Jul 15;14(7):858. doi: 10.3390/biology14070858 (PMC12292103; doi:10.3390/biology14070858)
Supplement: Supplementary file 1 [file biology-14-00858-s001.zip › Table S1-new.pdf]

Table S1 Detailed information of the 13 sequenced *Proteus vulgaris* strains used in this study.

**Strain name**	**Accession code**	**Isolation source**	**Geographic location**	**Collection date**	**Sequencing technology**	**Genome coverage**	**Assembly Method**	**Genome Representation**
Proteus vulgaris strain P3M	CP060211	*Penaeus vannamei*	China: Tianjin	10-06-2013	Illumina HiSeq	10.0x	HGAP v. 4.0	Full
Proteus vulgaris strain CCU063	CP032663	Blood	Taiwan	2015	Oxford Nanopore; Illumina	40.0x	Unicycler v. 0.4.7	Full
Proteus vulgaris strain FADDRGOS_366	CP150645	Pig	China: Heilongjiang province	04-02-2023	Illumina NovaSeq; Oxford Nanopore MinION	350.0x	Unicycler v. 0.4.3	Full
Proteus vulgaris strain FADDR-GOS_566	CP033736	Homo sapiens	--	--	PacBio; Illumina	1042.77348761905x	Canu v. 1.4	--
Proteus vulgaris strain FADDRGOS_1507	CP083628	--	Germany: Braunschweig	--	Pacbio; Illumina	1446.94x	SMRT v. 9.0.0, HGAP v. 4.0	--
Proteus vulgaris strain HH17	CP054157	--	--	2009	ONT Minion	145.2x	Shasta; Medaka v. Shasta v0.4.0; Medaka v0.11.5	Full
Proteus vulgaris strain LC-693	CP063314	Rectal swab	Italy	15-06-2020	Oxford Nanopore MinION;	520.0x	Unicycler v. v.0.4.8	Full

					Illumina NovaSeq			
Proteus vulgaris strain PvSC3	CP034668	Chicken	China: Mianyang	29-11-2017	Illumina MiSeq; PacBio RSII	100.0x	SMRT portal v. 3.2.0	Full
Proteus vulgaris strain TAF3	CP126335	Water	China: Jinan	16-05-2022	Oxford Nanopore; BGI	1026.0x	canu v. 1.5	Full
Proteus vulgaris strain USDA-ARS-USMARC-49741	CP104121	Feedlot	USA: Kansas	29-09-2013	PacBio RSII	201x	HGAP v. 3	Full
Proteus vulgaris strain ZN3	CP047344	A nose swab sample of swine origin	China: Nantong, jiangsu province	05-2018	Oxford Nanopore MiniION; Illumina HiSeq	50.0x	Unicycler v. v0.4.4	Full
Proteus vulgaris strain Ld01	CP090064	*Leptinotarsa decemlineata*	China: Nanjing	20-05-2021	Oxford Nanopore PromethION; BGISEQ	184.81x	unicycler v. SEPTEMBER-2021	Full
Proteus vulgaris strain 2023JQ-00005	CP137920	Wound	USA	2022	Oxford Nanopore GridION; Illumina MiSeq	102.0x	Unicycler v. 0.5.0	Full
