# Supplementary material for: Unveiling Genomic Islands Hosting Antibiotic Resistance Genes and Virulence Genes in Foodborne Multidrug-Resistant Patho-Genic Proteus vulgaris
Source: Biology (Basel). 2025 Jul 15;14(7):858. doi: 10.3390/biology14070858 (PMC12292103; doi:10.3390/biology14070858)
Supplement: Supplementary file 1 [file biology-14-00858-s001.zip › Table S2-revised.pdf]

Table S2 Summary of bioinformatics tools and databases used in this study.

Tool/Resource	Version	Access Mode	URL
IslandViewer4	4.0.1	Web-based	https://www.pathogenomics.sfu.ca/islandviewer/browse/
CARD-RGI	6.0.0	Command-line	https://card.mcmaster.ca/
IslandCompare	1.1.0	Web-based	https://islandcompare.ca/analysis?id=ff959500-555c-11ef-b9c1-059f1c7f7fe4
Easyfig	2.2.3	Local (Python)	https://github.com/mjsull/Easyfig
VFDB	2021	Web-based	https://www.mgc.ac.cn/VFs/main.htm
MEGA7	7.0.26	Local (GUI)	https://www.megasoftware.net/
