# Supplementary material for: Unveiling Genomic Islands Hosting Antibiotic Resistance Genes and Virulence Genes in Foodborne Multidrug-Resistant Patho-Genic Proteus vulgaris
Source: Biology (Basel). 2025 Jul 15;14(7):858. doi: 10.3390/biology14070858 (PMC12292103; doi:10.3390/biology14070858)
Supplement: Supplementary file 1 [file biology-14-00858-s001.zip › Table S3-revised.pdf]

Table S3 Potential antibiotic resistance genes (ARGs) on the P3M genome predicted by CARD database. CARD database predictions with parameters: ⩾80% identity, ⩾80% coverage.

**No.**	**CARD_Info**	**Drug_class**	**Resistance_mechanism**	**Gene_name**	**Description**
1	gb|WP_004236715.1|ARO:3003306|Morganella	fluoroquinolone antibiotic;aminocoumarin antibiotic	antibiotic target alteration	Morganella morganii gyrB conferring resistance to fluoroquinolone	Point mutation in Morganella morganii resulting in fluoroquinolone resistance
2	gb|AAC75089.1|ARO:3003577|ugd	peptide antibiotic	antibiotic target alteration	ugd	PmrE is required for the synthesis and transfer of 4-amino-4-deoxy-L-arabinose (Ara4N) to Lipid A, which allows gram-negative bacteria to resist the antimicrobial activity of cationic antimicrobial peptides and antibiotics such as polymyxin
3	gb|NP_312864.1|ARO:3000830|cpxA	aminoglycoside antibiotic;aminocoumarin antibiotic	antibiotic efflux	cpxA	CpxA is a membrane-localized sensor kinase that is activated by envelope stress. It starts a kinase cascade that activates CpxR, which promotes efflux complex expression.

4	gb|SIP52035.1|ARO:3004054|Pseudomonas	macrolide antibiotic;fluoroquinolone antibiotic;monobactam;aminoglycoside antibiotic;carbapenem;cephalosporin;cephamycin;penam;tetracycline antibiotic;peptide antibiotic;aminocoumarin antibiotic;diaminopyrimidine antibiotic;sulfonamide antibiotic;phenicol antibiotic;penem	antibiotic efflux	Pseudomonas aeruginosa CpxR	CpxR is directly involved in activation of expression of RND efflux pump MexAB-OprM in P. aeruginosa. CpxR is required to enhance mexAB-oprM expression and drug resistance, in the absence of repressor MexR.
5	gb|AAD22144.1|ARO:3002698|cmlB	phenicol antibiotic	antibiotic efflux	cmlB	cmlB is a plasmid-encoded chloramphenicol exporter that is found in Enterobacter aerogenes

6	gb|AAA50993.1|ARO:3003368|Escherichia	elfamycin antibiotic	antibiotic target alteration	Escherichia coli EF-Tu mutants conferring resistance to kirromycin	Sequence variants of Escherichia coli elongation factor Tu that confer resistance to kirromycin
7	gb|YP_208874.1|ARO:3003930|rpsJ	tetracycline antibiotic	antibiotic target protection	rpsJ	rpsJ is a tetracycline resistance protein identified in Neisseria gonorrhoeae. Tetracycline resistance is conferred by binding to the ribosome as a 30S ribosomal protection protein.
8	gb|ADL24064.1|ARO:3003737|Staphylococcus	fusidic acid	antibiotic target alteration	Staphylococcus aureus fusE with mutation conferring resistance to fusidic acid	The mutations to the rplF gene encoding riboprotein L6 have been shown to cause fusidic acid resistance, demonstrating a potential secondary site of action of the antibiotic that is blocked through these mutations. Several types of mutations have been identified that cause resistance, including SNPs, frameshift mutations and early stop codons.

9	gb|CDJ73082|ARO:3003900|Escherichia	fosfomycin	antibiotic target alteration	Escherichia coli CyaA with mutation conferring resistance to fosfomycin	CyaA (adenylate cyclase) is involved with the synthesis of cyclic AMP which regulates the fosfomycin transporter glpT. As a result, mutations to cyaA confer resistance to fosfomycin.
10	gb|WP_000764731.1|ARO:3003386|Escherichia	sulfonamide antibiotic;sulfone antibiotic	antibiotic target alteration	Escherichia coli folP with mutation conferring resistance to sulfonamides	Point mutations in Escherichia coli dihydropteroate synthase folP prevent sulfonamide antibiotics from inhibiting its role in folate synthesis, thus conferring sulfonamide resistance.
11	gb|AEY83581|ARO:3000510|mupB	mupirocin	antibiotic target alteration	Staphylococcus mupB conferring resistance to mupirocin	An alternative isoleucyl-tRNA synthetase conferring resistance to mupirocin.
12	gb|NP_816529.1|ARO:3003792|Enterococcus	peptide antibiotic	antibiotic target alteration	Enterococcus faecalis liaR mutant conferring daptomycin resistance	liaR is a response regulator found in the liaFSR signal transduction pathway. Mutations confer daptomycin resistance.

13	gb|AAO82600.1|ARO:3003791|Enterococcus	peptide antibiotic	antibiotic target alteration	Enterococcus faecalis liaS mutant conferring daptomycin resistance	liaS is a histidine kinase found in the liaFSR signal transduction pathway. Mutations confer daptomycin resistance.
14	gb|BAE78116.1|ARO:3003576|eptA	peptide antibiotic	antibiotic target alteration	eptA	PmrC mediates the modification of Lipid A by the addition of 4-amino-4-deoxy-L-arabinose (L-Ara4N) and phosphoethanolamine, resulting in a less negative cell membrane and decreased binding of polymyxin B.
15	gb|CDJ72593|ARO:3003889|Escherichia	fosfomycin	antibiotic target alteration	Escherichia coli GlpT with mutation conferring resistance to fosfomycin	Point mutations to the active importer GlpT, which is involved with the uptake of many phosphorylated sugars, confer resistance to fosfomycin by reducing import of the drug into the bacteria.

16	gb|AAV85982.1|ARO:3000535|macB	macrolide antibiotic	antibiotic efflux	macB	MacB is an ATP-binding cassette (ABC) transporter that exports macrolides with 14- or 15- membered lactones. It forms an antibiotic efflux complex with MacA and TolC. macB corresponds to 1 locus in Pseudomonas aeruginosa PAO1 and 1 locus in Pseudomonas aeruginosa LESB58.
17	gb|NP_415434.1|ARO:3003950|msbA	nitroimidazole antibiotic	antibiotic efflux	msbA	MsbA is a multidrug resistance transporter homolog from E. coli and belongs to a superfamily of transporters that contain an adenosine triphosphate (ATP) binding cassette (ABC) which is also called a nucleotide-binding domain (NBD). MsbA is a member of the MDR-ABC transporter group by sequence homology. MsbA transports lipid A, a major component of the bacterial outer cell membrane, and is the only bacterial ABC transporter that is essential for cell viability.

18	gb|APB03214.1|ARO:3003980|tetA(48)	tetracycline antibiotic	antibiotic efflux	tetA(48)	TetA(48) is a Tetracycline efflux pump described in Paenibacillus sp. LC231, a strain of Paenibacillus isolated from Lechuguilla Cave, NM, USA. Described by Pawlowski et al. 2016.
19	gb|AAA26832.1|ARO:3002827|tlrC	macrolide antibiotic	antibiotic target protection	tlrC	tlrC is an efflux pump found in Streptomyces fradiae and confers resistance to mycinamicin, tylosin and lincosamides. tlrC is found in the tylosin biosynthetic cluster and is one mechanism by which S. fradiae protects itself from self-destruction when producing this macrolide.

20	gb|AIL15701|ARO:3003775|Escherichia	fosfomycin	antibiotic target alteration	Escherichia coli murA with mutation conferring resistance to fosfomycin	murA or UDP-N-acetylglucosamine enolpyruvyl transferase catalyses the initial step in peptidoglycan biosynthesis and is inhibited by fosfomycin. Overexpression of murA through mutations such as Asp369Asn and Leu370Ile confers fosfomycin resistance. Extensive evidence has shown the significance of C115 mutations in conferring fosfomycin resistance since this residue represents a primary binding site for the antibiotic across many species.
21	gb|AAC75429.1|ARO:3000833|evgS	macrolide antibiotic;fluoroquinolone antibiotic;penam;tetracycline antibiotic	antibiotic efflux	evgS	EvgS is a sensor protein that phosphorylates the regulatory protein EvgA. evgS corresponds to 1 locus in Pseudomonas aeruginosa PAO1 and 1 locus in Pseudomonas aeruginosa LESB58.
22	gb|CDO61513.1|ARO:3003948|efrA	macrolide antibiotic;fluoroquinolone antibiotic;rifamycin antibiotic	antibiotic efflux	efrA	efrA is a part of the EfrAB efflux pump, and both efrA and efrB are necessary to confer drug resistance.

23	gb|APB03219.1|ARO:3003986|TaeA	pleuromutilin antibiotic	antibiotic efflux	TaeA	Pleuromutilin (Tiamulin) ABC efflux pump found in Paenibacillus sp. LC231, a strain of Paenibacillus isolated from Lechuguilla Cave, NM, USA. Confers resistance to pleuromutilin antibiotics. Described by Pawlowski et al. 2016.
24	gb|CAE51745.1|ARO:3000168|tet(D)	tetracycline antibiotic	antibiotic efflux	tet(D)	TetD is a tetracycline efflux pump found exclusively in Gram-negative bacteria.
25	gb|AAR84672.1|ARO:3002925|vanRF	glycopeptide antibiotic	antibiotic target alteration	vanRF	vanRF is a vanR variant found in the vanF gene cluster
26	gb|CAA52296.1|ARO:3003729|Staphylococcus	mupirocin	antibiotic target alteration	Staphylococcus aureus ileS with mutation conferring resistance to mupirocin	Point mutations to the isoleucyl-tRNA synthetase (ileS) in Staphylococcus aureus that confer resistance to mupirocin.
27	gb|CCP46065.1|ARO:3000816|mtrA	macrolide antibiotic;penam	antibiotic efflux	mtrA	MtrA is a transcriptional activator of the MtrCDE multidrug efflux pump of Neisseria gonorrhoeae.

28	gb|CAG39047|ARO:3003794|Staphylococcus	peptide antibiotic	antibiotic target alteration	Staphylococcus aureus walK with mutation conferring resistance to daptomycin	walK is the histidine kinase sensor of a two-component regulatory system controlling peptidoglycan metabolism through regulation of the expression of most of the peptidoglycan hydrolase genes. Mutations in the gene have been found that confer daptomycin resistance.
29	gb|ABF66011.1|ARO:3002881|lmrC	lincosamide antibiotic	antibiotic target protection	lmrC	lmrC is a chromosomally-encoded efflux pump that confers resistance to lincosamides in Streptomyces lincolnensis and Lactococcus lactis. It can dimerize with lmrD
30	gb|NP_207972.1|ARO:3003964|hp1181	fluoroquinolone antibiotic;tetracycline antibiotic;nitroimidazole antibiotic	antibiotic efflux	hp1181	hp1181 is a translocase that is part of the MFS efflux pump family. It is found in H. pylori and has role in the active efflux of antibiotics.
31	gb|ANZ79240.1|ARO:3004035|tetA(60)	tetracycline antibiotic	antibiotic efflux	tetA(60)	tetA(60) is a subunit of tetAB(60), an ABC transporter that confers resistance to tetracycline and tigercycline identified by screening a human saliva metagenomic library in Escherichia coli, which is required for resistance.

32	gb|ANZ79241.1|ARO:3004036|tetB(60)	tetracycline antibiotic	antibiotic efflux	tetB(60)	tetB(60) is a subunit of tetAB(60), an ABC transporter that confers resistance to tetracycline and tigercycline identified by screening a human saliva metagenomic library in Escherichia coli, which is required for resistance.
33	gb|NP_414995.1|ARO:3000216|acrB	fluoroquinolone antibiotic;cephalosporin;glycylcycline;penam;tetracycline antibiotic;rifamycin antibiotic;phenicol antibiotic;triclosan	antibiotic efflux	acrB	Protein subunit of AcrA-AcrB-TolC multidrug efflux complex. AcrB functions as a herterotrimer which forms the inner membrane component and is primarily responsible for substrate recognition and energy transduction by acting as a drug/proton antiporter.

34	gb|NP_414996.1|ARO:3004043|Escherichia	fluoroquinolone antibiotic;cephalosporin;glycylcycline;penam;tetracycline antibiotic;rifamycin antibiotic;phenicol antibiotic;triclosan	antibiotic efflux	Escherichia coli acrA	AcrA is a subunit of the AcrAB-TolC multidrug efflux system that in E. coli.
35	gb|NP_414997.1|ARO:3003807|Escherichia	fluoroquinolone antibiotic;cephalosporin;glycylcycline;penam;tetracycline antibiotic;rifamycin antibiotic;phenicol antibiotic;triclosan	antibiotic target alteration;antibiotic efflux	Escherichia coli acrR with mutation conferring multidrug antibiotic resistance	AcrR is a repressor of the AcrAB-TolC multidrug efflux complex. AcrR mutations result in high level antibiotic resistance. The mutations associated with this model are specific to E. coli.
36	gb|APB03214.1|ARO:3003980|tetA(48)	tetracycline antibiotic	antibiotic efflux	tetA(48)	TetA(48) is a Tetracycline efflux pump described in Paenibacillus sp. LC231, a strain of Paenibacillus isolated from Lechuguilla Cave, NM, USA. Described by Pawlowski et al. 2016.

37	gb|CAA79966.1|ARO:3003665|NmcR	carbapenem;cephalosporin;cephamycin;penam	antibiotic inactivation	NmcR	NmcR is a homolog of the LysR regulator found in Enterobacter cloacae that contribute to the regulation of NmcA beta-lactamase
38	gb|AAV85982.1|ARO:3000535|macB	macrolide antibiotic	antibiotic efflux	macB	MacB is an ATP-binding cassette (ABC) transporter that exports macrolides with 14- or 15- membered lactones. It forms an antibiotic efflux complex with MacA and TolC. macB corresponds to 1 locus in Pseudomonas aeruginosa PAO1 and 1 locus in Pseudomonas aeruginosa LESB58.
39	gb|CDJ73208|ARO:3003890|Escherichia	fosfomycin	antibiotic target alteration	Escherichia coli UhpT with mutation conferring resistance to fosfomycin	Mutations to the active importer UhpT, which is involved with the uptake of many phosphorylated sugars, confer resistance to fosfomycin by reducing import of the drug into the bacteria.

40	gb|CDJ73205|ARO:3003893|Escherichia	fosfomycin	antibiotic target alteration	Escherichia coli UhpA with mutation conferring resistance to fosfomycin	uhpA is a positive activator of the fosfomycin importer uhpT, thus mutations to uhpA confer fosfomycin resistance by reducing uhpT expression. Both knockout and amino acid substitution mutations have been found that confer resistance, with the Protein Knockout model describing the large, knockout mutations causing loss of function of the gene, and the Protein Variant model describing the amino acid substitutions.
41	gb|AFC91828.1|ARO:3003041|Streptococcus	monobactam;carbapenem;cephalosporin;cephamycin;penam	antibiotic target alteration	Streptococcus pneumoniae PBP1a conferring resistance to amoxicillin	PBP1a is a penicillin-binding protein found in Streptococcus pneumoniae
42	gb|WP_001283444.1|ARO:3000815|mgrA	fluoroquinolone antibiotic;cephalosporin;penam;tetracycline antibiotic;peptide antibiotic;acridine dye	antibiotic efflux	mgrA	MgrA, also known as NorR, is a regulator for norA, norB, and tet38. It is a positive regulator for norA expression, but is a direct repressor for norB and an indirect repressor of tet38.

43	gb|NP_253661.1|ARO:3003682|OpmH	triclosan	antibiotic efflux	OpmH	OpmH is an outer membrane efflux protein required for triclosan-specific efflux pump function.
44	gb|CDO61516.1|ARO:3003949|efrB	macrolide antibiotic;fluoroquinolone antibiotic;rifamycin antibiotic	antibiotic efflux	efrB	efrB is a part of the EfrAB efflux pump, and both efrA and efrB are necessary to confer multidrug resistance.
45	gb|AAC75733.1|ARO:3000074|emrB	fluoroquinolone antibiotic	antibiotic efflux	emrB	emrB is a translocase in the emrB -TolC efflux protein in E. coli. It recognizes substrates including carbonyl cyanide m-chlorophenylhydrazone (CCCP), nalidixic acid, and thioloactomycin.
46	gb|BAA16547.1|ARO:3000027|emrA	fluoroquinolone antibiotic	antibiotic efflux	emrA	EmrA is a membrane fusion protein, providing an efflux pathway with EmrB and TolC between the inner and outer membranes of E. coli, a Gram-negative bacterium.
47	gb|NP_417169.1|ARO:3000516|emrR	fluoroquinolone antibiotic	antibiotic efflux	emrR	EmrR is a negative regulator for the EmrAB-TolC multidrug efflux pump in E. coli. Mutations lead to EmrAB-TolC overexpression.

48	gb|ABF66011.1|ARO:3002881|lmrC	lincosamide antibiotic	antibiotic target protection	lmrC	lmrC is a chromosomally-encoded efflux pump that confers resistance to lincosamides in Streptomyces lincolnensis and Lactococcus lactis. It can dimerize with lmrD
49	gb|WP_000725529.1|ARO:3001209|mecC	monobactam;carbapenem;cephalosporin;cephamycin;penam	antibiotic target replacement	mecC	A foreign PBP2a acquired by lateral gene transfer that able to perform peptidoglycan synthesis in the presence of beta-lactams.
50	gb|AEY83581|ARO:3000510|mupB	mupirocin	antibiotic target alteration	Staphylococcus mupB conferring resistance to mupirocin	An alternative isoleucyl-tRNA synthetase conferring resistance to mupirocin.
51	gb|AAV85982.1|ARO:3000535|macB	macrolide antibiotic	antibiotic efflux	macB	MacB is an ATP-binding cassette (ABC) transporter that exports macrolides with 14- or 15- membered lactones. It forms an antibiotic efflux complex with MacA and TolC. macB corresponds to 1 locus in Pseudomonas aeruginosa PAO1 and 1 locus in Pseudomonas aeruginosa LESB58.

52	gb|APB03219.1|ARO:3003986|TaeA	pleuromutilin antibiotic	antibiotic efflux	TaeA	Pleuromutilin (Tiamulin) ABC efflux pump found in Paenibacillus sp. LC231, a strain of Paenibacillus isolated from Lechuguilla Cave, NM, USA. Confers resistance to pleuromutilin antibiotics. Described by Pawlowski et al. 2016.
53	gb|NP_415434.1|ARO:3003950|msbA	nitroimidazole antibiotic	antibiotic efflux	msbA	MsbA is a multidrug resistance transporter homolog from E. coli and belongs to a superfamily of transporters that contain an adenosine triphosphate (ATP) binding cassette (ABC) which is also called a nucleotide-binding domain (NBD). MsbA is a member of the MDR-ABC transporter group by sequence homology. MsbA transports lipid A, a major component of the bacterial outer cell membrane, and is the only bacterial ABC transporter that is essential for cell viability.

54	gb|APB03214.1|ARO:3003980|tetA(48)	tetracycline antibiotic	antibiotic efflux	tetA(48)	TetA(48) is a Tetracycline efflux pump described in Paenibacillus sp. LC231, a strain of Paenibacillus isolated from Lechuguilla Cave, NM, USA. Described by Pawlowski et al. 2016.
55	gb|NP_273367.1|ARO:3003961|farA	antibacterial free fatty acids	antibiotic efflux	farA	farA is the membrane fusion protein that is part of the farAB efflux pump.
56	gb|NP_415434.1|ARO:3003950|msbA	nitroimidazole antibiotic	antibiotic efflux	msbA	MsbA is a multidrug resistance transporter homolog from E. coli and belongs to a superfamily of transporters that contain an adenosine triphosphate (ATP) binding cassette (ABC) which is also called a nucleotide-binding domain (NBD). MsbA is a member of the MDR-ABC transporter group by sequence homology. MsbA transports lipid A, a major component of the bacterial outer cell membrane, and is the only bacterial ABC transporter that is essential for cell viability.

57	gb|AAV85981.1|ARO:3000533|macA	macrolide antibiotic	antibiotic efflux	macA	MacA is a membrane fusion protein that forms an antibiotic efflux complex with MacB and TolC. macA corresponds to 1 locus in Pseudomonas aeruginosa PAO1 and 1 locus in Pseudomonas aeruginosa LESB58.
58	gb|AAV85982.1|ARO:3000535|macB	macrolide antibiotic	antibiotic efflux	macB	MacB is an ATP-binding cassette (ABC) transporter that exports macrolides with 14- or 15- membered lactones. It forms an antibiotic efflux complex with MacA and TolC. macB corresponds to 1 locus in Pseudomonas aeruginosa PAO1 and 1 locus in Pseudomonas aeruginosa LESB58.

59	gb|AAV85982.1|ARO:3000535|macB	macrolide antibiotic	antibiotic efflux	macB	MacB is an ATP-binding cassette (ABC) transporter that exports macrolides with 14- or 15- membered lactones. It forms an antibiotic efflux complex with MacA and TolC. macB corresponds to 1 locus in Pseudomonas aeruginosa PAO1 and 1 locus in Pseudomonas aeruginosa LESB58.
60	gb|NP_415434.1|ARO:3003950|msbA	nitroimidazole antibiotic	antibiotic efflux	msbA	MsbA is a multidrug resistance transporter homolog from E. coli and belongs to a superfamily of transporters that contain an adenosine triphosphate (ATP) binding cassette (ABC) which is also called a nucleotide-binding domain (NBD). MsbA is a member of the MDR-ABC transporter group by sequence homology. MsbA transports lipid A, a major component of the bacterial outer cell membrane, and is the only bacterial ABC transporter that is essential for cell viability.

61	gb|NP_415434.1|ARO:3003950|msbA	nitroimidazole antibiotic	antibiotic efflux	msbA	MsbA is a multidrug resistance transporter homolog from E. coli and belongs to a superfamily of transporters that contain an adenosine triphosphate (ATP) binding cassette (ABC) which is also called a nucleotide-binding domain (NBD). MsbA is a member of the MDR-ABC transporter group by sequence homology. MsbA transports lipid A, a major component of the bacterial outer cell membrane, and is the only bacterial ABC transporter that is essential for cell viability.

62	gb|NP_415434.1|ARO:3003950|msbA	nitroimidazole antibiotic	antibiotic efflux	msbA	MsbA is a multidrug resistance transporter homolog from E. coli and belongs to a superfamily of transporters that contain an adenosine triphosphate (ATP) binding cassette (ABC) which is also called a nucleotide-binding domain (NBD). MsbA is a member of the MDR-ABC transporter group by sequence homology. MsbA transports lipid A, a major component of the bacterial outer cell membrane, and is the only bacterial ABC transporter that is essential for cell viability.

63	gb|AIA49384.1|ARO:3004128|Serratia	fluoroquinolone antibiotic;monobactam;carbapenem;cephalosporin;cephamycin;penam;tetracycline antibiotic;phenicol antibiotic;penem	reduced permeability to antibiotic;resistance by absence	Serratia marcescens Omp1	Omp1 is an outer membrane porin that confers resistance by absence in S. marcescens. Knockout, deletion or other inhibition of the omp1 gene confers resistance to certain beta-lactamase antibiotics - including Cefoxitin, Ceftriaxone, Cefotaxime, and Moxalactam - as well as Ciprofloxacin, Tetracycline, and Chloramphenicol, by preventing passage of the antibiotic into the cell.
64	gb|APB03219.1|ARO:3003986|TaeA	pleuromutilin antibiotic	antibiotic efflux	TaeA	Pleuromutilin (Tiamulin) ABC efflux pump found in Paenibacillus sp. LC231, a strain of Paenibacillus isolated from Lechuguilla Cave, NM, USA. Confers resistance to pleuromutilin antibiotics. Described by Pawlowski et al. 2016.
65	gb|BAE78083.1|ARO:3003549|mdtO	nucleoside antibiotic;acridine dye	antibiotic efflux	mdtO	Multidrug resistance efflux pump. Could be involved in resistance to puromycin, acriflavine and tetraphenylarsonium chloride

66	gb|BAE78084.1|ARO:3003548|mdtN	nucleoside antibiotic;acridine dye	antibiotic efflux	mdtN	Multidrug resistance efflux pump. Could be involved in resistance to puromycin, acriflavine and tetraphenylarsonium chloride.
67	gb|AFK13828.1|ARO:3000823|ramA	fluoroquinolone antibiotic;monobactam;carbapenem;cephalosporin;glycylcycline;cephamycin;penam;tetracycline antibiotic;rifamycin antibiotic;phenicol antibiotic;triclosan;penem	antibiotic efflux;reduced permeability to antibiotic	ramA	RamA (resistance antibiotic multiple) is a positive regulator of AcrAB-TolC and leads to high level multidrug resistance in Klebsiella pneumoniae, Salmonella enterica, and Enterobacter aerugenes, increasing the expression of both the mar operon as well as AcrAB. RamA also decreases OmpF expression.
68	gb|YP_490321.1|ARO:3000828|baeR	aminoglycoside antibiotic;aminocoumarin antibiotic	antibiotic efflux	baeR	BaeR is a response regulator that promotes the expression of MdtABC and AcrD efflux complexes.
69	gb|BAA15934.1|ARO:3000829|baeS	aminoglycoside antibiotic;aminocoumarin antibiotic	antibiotic efflux	baeS	BaeS is a sensor kinase in the BaeSR regulatory system. While it phosphorylates BaeR to increase its activity, BaeS is not necessary for overexpressed BaeR to confer resistance.

70	gb|AAC75137.1|ARO:3000794|mdtC	aminocoumarin antibiotic	antibiotic efflux	mdtC	MdtC is a transporter that forms a heteromultimer complex with MdtB to form a multidrug transporter. MdtBC is part of the MdtABC-TolC efflux complex. In the absence of MdtB, MdtC can form a homomultimer complex that results in a functioning efflux complex with a narrower drug specificity. mdtC corresponds to 3 loci in Pseudomonas aeruginosa PAO1 (gene name: muxC/muxB) and 3 loci in Pseudomonas aeruginosa LESB58.
71	gb|AAC75136.1|ARO:3000793|mdtB	aminocoumarin antibiotic	antibiotic efflux	mdtB	MdtB is a transporter that forms a heteromultimer complex with MdtC to form a multidrug transporter. MdtBC is part of the MdtABC-TolC efflux complex.
72	gb|AAC75135.2|ARO:3000792|mdtA	aminocoumarin antibiotic	antibiotic efflux	mdtA	MdtA is the membrane fusion protein of the multidrug efflux complex mdtABC.

73	gb|AFK58562.1|ARO:3003078|Enterococcus	peptide antibiotic	antibiotic target alteration	Enterococcus faecium liaR mutant conferring daptomycin resistance	liaR is a response regulator found in the liaFSR signal transduction pathway. Mutations confer daptomycin resistance.
74	gb|AAV85982.1|ARO:3000535|macB	macrolide antibiotic	antibiotic efflux	macB	MacB is an ATP-binding cassette (ABC) transporter that exports macrolides with 14- or 15- membered lactones. It forms an antibiotic efflux complex with MacA and TolC. macB corresponds to 1 locus in Pseudomonas aeruginosa PAO1 and 1 locus in Pseudomonas aeruginosa LESB58.
75	gb|NP_415611.1|ARO:3004049|antibiotic	triclosan	antibiotic target alteration	Escherichia coli fabG mutations conferring resistance to triclosan	fabG is a 3-oxoacyl-acyl carrier protein reductase involved in lipid metabolism and fatty acid biosynthesis.The bacterial biocide Triclosan blocks the final reduction step in fatty acid elongation, inhibiting biosynthesis. Point mutations in fabG can confer resistance to Triclosan.

76	gb|WP_001025093|ARO:3003323|Staphylococcus	peptide antibiotic	antibiotic target alteration	Staphylococcus aureus pgsA mutations conferring resistance to daptomycin	Point mutations that occur within Staphylococcus aureus pgsA gene resulting in resistance to daptomycin
77	gb|CAA42594.1|ARO:3002690|cml	phenicol antibiotic	antibiotic efflux	Streptomyces lividans cmlR/satB	cmlR/satB is a plasmid or chromosome-encoded chloramphenicol resistance determinant (putative transmembrane protein) that is found in Escherichia coli and Streptomyces lividans
78	gb|CAA77936.1|ARO:3004039|Escherichia	macrolide antibiotic	antibiotic efflux	Escherichia coli emrE	Member of the small MDR (multidrug resistance) family of transporters; in Escherichia coli this protein provides resistance against a number of positively charged compounds including ethidium bromide and erythromycin; proton-dependent secondary transporter which exchanges protons for compound translocation
79	gb|ABA71733.1|ARO:3002972|vanTG	glycopeptide antibiotic	antibiotic target alteration	vanTG	vanTG is a vanT variant found in the vanG gene cluster

80	gb|NP_310518|ARO:3004127|Escherichia	fluoroquinolone antibiotic;aminoglycoside antibiotic	reduced permeability to antibiotic;resistance by absence	Escherichia coli mipA	MltA-interacting protein (mipA), is an antibiotic resistance-related outer membrane protein. Deletion of mipA increases kanamycin, nalidixic acid and streptomycin resistance.
81	gb|CCP44816.1|ARO:3003394|Mycobacterium	pyrazinamide	antibiotic target alteration	Mycobacterium tuberculosis pncA mutations conferring resistance to pyrazinamide	pncA is a pyrazinamidase/nicotinamidase. It catalyzes the activation of pyrazinamide. Some mutation within pncA are associated with loss of enzyme activity, resulting in pyrazinamide resistance.
82	gb|NP_309766.1|ARO:3000676|H-NS	macrolide antibiotic;fluoroquinolone antibiotic;cephalosporin;cephamycin;penam;tetracycline antibiotic	antibiotic efflux	H-NS	H-NS is a histone-like protein involved in global gene regulation in Gram-negative bacteria. It is a repressor of the membrane fusion protein genes acrE, mdtE, and emrK as well as nearby genes of many RND-type multidrug exporters.
83	gb|AAA99504.1|ARO:3002987|bcrA	peptide antibiotic	antibiotic efflux	bcrA	bcrA is an ABC transporter found in Bacillus licheniformis that confers bacitracin resistance

84	gb|APB03214.1|ARO:3003980|tetA(48)	tetracycline antibiotic	antibiotic efflux	tetA(48)	TetA(48) is a Tetracycline efflux pump described in Paenibacillus sp. LC231, a strain of Paenibacillus isolated from Lechuguilla Cave, NM, USA. Described by Pawlowski et al. 2016.
85	gb|AAF67494.2|ARO:3002522|novA	aminocoumarin antibiotic	antibiotic efflux	novA	A type III ABC transporter, identified on the novobiocin biosynthetic gene cluster, involved in the transport and resistance of novobiocin.
86	gb|AAL78278.2|ARO:3002401|OXY-2-6	monobactam;cephalosporin;penam	antibiotic inactivation	OXY-2-6	OXY-2-6 is a beta-lactamase found in Klebsiella oxytoca
87	gb|CAA79966.1|ARO:3003665|NmcR	carbapenem;cephalosporin;cephamycin;penam	antibiotic inactivation	NmcR	NmcR is a homolog of the LysR regulator found in Enterobacter cloacae that contribute to the regulation of NmcA beta-lactamase

88	gb|NP_416715.1|ARO:3003952|yojI	peptide antibiotic	antibiotic efflux	YojI	YojI mediates resistance to the peptide antibiotic microcin J25 when it is expressed from a multicopy vector. YojI is capable of pumping out microcin molecules. The outer membrane protein TolC in addition to YojI is required for export of microcin J25 out of the cell. Microcin J25 is thus the first known substrate for YojI.
89	gb|NP_416715.1|ARO:3003952|yojI	peptide antibiotic	antibiotic efflux	YojI	YojI mediates resistance to the peptide antibiotic microcin J25 when it is expressed from a multicopy vector. YojI is capable of pumping out microcin molecules. The outer membrane protein TolC in addition to YojI is required for export of microcin J25 out of the cell. Microcin J25 is thus the first known substrate for YojI.
90	gb|AAC74149.2|ARO:3001216|mdtH	fluoroquinolone antibiotic	antibiotic efflux	mdtH	Multidrug resistance protein MdtH
91	gb|AAT46077.1|ARO:3000300|lsaA	lincosamide antibiotic;streptogramin antibiotic;pleuromutilin antibiotic	antibiotic target protection	lsaA	LsaA is an ABC efflux pump expressed in Enterococcus faecalis. It confers resistance to clindamycin, quinupristin-dalfopristin, and dalfopristin.

92	gb|AAA99504.1|ARO:3002987|bcrA	peptide antibiotic	antibiotic efflux	bcrA	bcrA is an ABC transporter found in Bacillus licheniformis that confers bacitracin resistance
93	gb|AAZ42322.1|ARO:3003836|qacH	fluoroquinolone antibiotic	antibiotic efflux	qacH	qacH is a subunit of the qac multidrug efflux pump in Vibrio cholerae
94	gb|ABF66011.1|ARO:3002881|lmrC	lincosamide antibiotic	antibiotic target protection	lmrC	lmrC is a chromosomally-encoded efflux pump that confers resistance to lincosamides in Streptomyces lincolnensis and Lactococcus lactis. It can dimerize with lmrD
95	gb|CAE51745.1|ARO:3000168|tet(D)	tetracycline antibiotic	antibiotic efflux	tet(D)	TetD is a tetracycline efflux pump found exclusively in Gram-negative bacteria.

96	gb|NP_252264.1|ARO:3000819|nalD	macrolide antibiotic;fluoroquinolone antibiotic;monobactam;carbapenem;cephalosporin;cephamycin;penam;tetracycline antibiotic;peptide antibiotic;aminocoumarin antibiotic;diaminopyrimidine antibiotic;sulfonamide antibiotic;phenicol antibiotic;penem	antibiotic efflux	nalD	NalD is a repressor of MexAB-OprM. Mutations lead to multidrug resistance and MexAB-OprM overexpression.
97	gb|AHA41499.1|ARO:3002948|vanHO	glycopeptide antibiotic	antibiotic target alteration	vanHO	vanHO is a vanH variant in the vanO gene cluster
98	gb|CAA37806.1|ARO:3002684|catII	phenicol antibiotic	antibiotic inactivation	catII/catA	catII/catA is a plasmid-encoded variant of the cat gene found in Haemophilus influenzae, Agrobacterium tumefaciens and Escherichia coli

99	gb|AAA99504.1|ARO:3002987|bcrA	peptide antibiotic	antibiotic efflux	bcrA	bcrA is an ABC transporter found in Bacillus licheniformis that confers bacitracin resistance
100	gb|NP_415222.1|ARO:3003841|kdpE	aminoglycoside antibiotic	antibiotic efflux	kdpE	kdpE is a transcriptional activator that is part of the two-component system KdpD/KdpE that is studied for its regulatory role in potassium transport and has been identified as an adaptive regulator involved in the virulence and intracellular survival of pathogenic bacteria. kdpE regulates a range of virulence loci through direct promoter binding.
101	gb|AAD51347.1|ARO:3003067|smeS	aminoglycoside antibiotic;cephalosporin;cephamycin;penam	antibiotic efflux	smeS	smeS is the protein kinase sensor component of a two component signal transduction system that includes smeR

102	gb|AAN62561.1|ARO:3003970|D-Ala-D-Ala	glycopeptide antibiotic	antibiotic target alteration	D-Ala-D-Ala ligase	Non-van ligases that synthesize D-Ala-D-Ala, the default cell wall precursor that makes a cell vulnerable to glycopeptide antibiotics. Mutations in the ddl gene can cause the production of nonfunctional/inactivated D-Ala-D-Ala ligases, which can render bacteria glycopeptide dependent depending on the presence of vancomycin resistance clusters.
103	gb|ALH22601.1|ARO:3000620|adeL	fluoroquinolone antibiotic;tetracycline antibiotic	antibiotic efflux	adeL	AdeL is a regulator of AdeFGH in Acinetobacter baumannii. AdeL mutations are associated with AdeFGH overexpression and multidrug resistance.
104	gb|WP_032492277.1|ARO:3004103|QepA2	fluoroquinolone antibiotic	antibiotic efflux	QepA2	QepA2 is a plasmid-mediated quinolone resistance pump found in an Escherichia coli isolate from France
105	gb|NP_252368.1|ARO:3003710|mexL	macrolide antibiotic;tetracycline antibiotic;triclosan	antibiotic efflux	MexL	MexL is a specific repressor of mexJK transcription and autoregulates its own expression.

106	gb|NP_415434.1|ARO:3003950|msbA	nitroimidazole antibiotic	antibiotic efflux	msbA	MsbA is a multidrug resistance transporter homolog from E. coli and belongs to a superfamily of transporters that contain an adenosine triphosphate (ATP) binding cassette (ABC) which is also called a nucleotide-binding domain (NBD). MsbA is a member of the MDR-ABC transporter group by sequence homology. MsbA transports lipid A, a major component of the bacterial outer cell membrane, and is the only bacterial ABC transporter that is essential for cell viability.
107	gb|AKA86814|ARO:3003746|optrA	oxazolidinone antibiotic	antibiotic target protection	optrA	optrA encodes an ABC-transporter gene conferring resistance to oxazolidinones that was isolated from a plasmid in Enterococcus faecalis and Enterococcus faecium.
108	gb|BAE78084.1|ARO:3003548|mdtN	nucleoside antibiotic;acridine dye	antibiotic efflux	mdtN	Multidrug resistance efflux pump. Could be involved in resistance to puromycin, acriflavine and tetraphenylarsonium chloride.

109	gb|AQX36338.1|ARO:3004379|QepA4	N/A	antibiotic efflux	QepA4	A plasmid-mediate quinolone efflux pump variant described in Escherichia coli. QepA4 confers resistance to quinolone and fluoroquinolone antibiotics by expulsion from the cell. Described by Manageiro et al. 2017.
110	gb|YP_500802.1|ARO:3003074|Staphylococcus	peptide antibiotic	antibiotic target alteration	Staphylococcus aureus cls conferring resistance to daptomycin	cls or cardiolipin synthetase is an inner membrane protein that is involved in membrane synthesis. Specific mutations in S. aureus can confer resistance to daptomycin.
111	gb|AAV85982.1|ARO:3000535|macB	macrolide antibiotic	antibiotic efflux	macB	MacB is an ATP-binding cassette (ABC) transporter that exports macrolides with 14- or 15- membered lactones. It forms an antibiotic efflux complex with MacA and TolC. macB corresponds to 1 locus in Pseudomonas aeruginosa PAO1 and 1 locus in Pseudomonas aeruginosa LESB58.

112	gb|ANZ79241.1|ARO:3004036|tetB(60)	tetracycline antibiotic	antibiotic efflux	tetB(60)	tetB(60) is a subunit of tetAB(60), an ABC transporter that confers resistance to tetracycline and tigercycline identified by screening a human saliva metagenomic library in Escherichia coli, which is required for resistance.
113	gb|NP_415804.1|ARO:3004045|antibiotic	isoniazid;triclosan	antibiotic target alteration	Escherichia coli fabI mutations conferring resistance to isoniazid and triclosan	fabI is a enoyl-acyl carrier reductase used in lipid metabolism and fatty acid biosynthesis. The bacterial biocide Triclosan blocks the final reduction step in fatty acid elongation, inhibiting biosynthesis. Point mutations in fabI can confer resistance to Triclosan and Isoniazid
114	gb|AAG16656.1|ARO:3002705|floR	phenicol antibiotic	antibiotic efflux	floR	floR is a plasmid or chromosome-encoded chloramphenicol exporter that is found in Bordetella bronchiseptica, Escherichia coli, Klebsiella pneumoniae,
115	gb|WP_014550864.1|ARO:3003953|hmrM	fluoroquinolone antibiotic;acridine dye	antibiotic efflux	hmrM	hmrM is a multidrug efflux pump belonging to the MATE family and functions as a Na+/drug antiporter.

116	gb|ABF66027.1|ARO:3002882|lmrD	lincosamide antibiotic	antibiotic efflux	lmrD	lmrD is a chromosomally-encoded efflux pump that confers resistance to lincosamides in Streptomyces lincolnensis and Lactococcus lactis. It can dimerize with lmrC
117	gb|APB03222.1|ARO:3003992|rphB	rifamycin antibiotic	antibiotic inactivation	rphB	rphB is a rifampin phosphotransferase protein found in Paenibacillus sp. LC231, a strain of Paenibacillus isolated from Lechuguilla Cave, NM, USA. Confers resistance to rifamycin antibiotics, specifically rifampin, through rifampin inactivation. Described by Pawlowski et al. 2016.
118	gb|AAV85982.1|ARO:3000535|macB	macrolide antibiotic	antibiotic efflux	macB	MacB is an ATP-binding cassette (ABC) transporter that exports macrolides with 14- or 15- membered lactones. It forms an antibiotic efflux complex with MacA and TolC. macB corresponds to 1 locus in Pseudomonas aeruginosa PAO1 and 1 locus in Pseudomonas aeruginosa LESB58.

119	gb|AFK13828.1|ARO:3000823|ramA	fluoroquinolone antibiotic;monobactam;carbapenem;cephalosporin;glycylcycline;cephamycin;penam;tetracycline antibiotic;rifamycin antibiotic;phenicol antibiotic;triclosan;penem	antibiotic efflux;reduced permeability to antibiotic	ramA	RamA (resistance antibiotic multiple) is a positive regulator of AcrAB-TolC and leads to high level multidrug resistance in Klebsiella pneumoniae, Salmonella enterica, and Enterobacter aerugenes, increasing the expression of both the mar operon as well as AcrAB. RamA also decreases OmpF expression.
120	gb|NP_252244|ARO:3002985|arnA	peptide antibiotic	antibiotic target alteration	arnA	arnA modifies lipid A with 4-amino-4-deoxy-L-arabinose (Ara4N) which allows gram-negative bacteria to resist the antimicrobial activity of cationic antimicrobial peptides and antibiotics such as polymyxin. arnA is found in E. coli and P. aeruginosa.

121	gb|AAC75314.1|ARO:3003578|pmrF	peptide antibiotic	antibiotic target alteration	PmrF	PmrF is required for the synthesis and transfer of 4-amino-4-deoxy-L-arabinose (Ara4N) to Lipid A, which allows gram-negative bacteria to resist the antimicrobial activity of cationic antimicrobial peptides and antibiotics such as polymyxin. pmrF corresponds to 1 locus in Pseudomonas aeruginosa PAO1 and 1 locus in Pseudomonas aeruginosa LESB58.
122	gb|APB03219.1|ARO:3003986|TaeA	pleuromutilin antibiotic	antibiotic efflux	TaeA	Pleuromutilin (Tiamulin) ABC efflux pump found in Paenibacillus sp. LC231, a strain of Paenibacillus isolated from Lechuguilla Cave, NM, USA. Confers resistance to pleuromutilin antibiotics. Described by Pawlowski et al. 2016.

123	gb|AAV85982.1|ARO:3000535|macB	macrolide antibiotic	antibiotic efflux	macB	MacB is an ATP-binding cassette (ABC) transporter that exports macrolides with 14- or 15- membered lactones. It forms an antibiotic efflux complex with MacA and TolC. macB corresponds to 1 locus in Pseudomonas aeruginosa PAO1 and 1 locus in Pseudomonas aeruginosa LESB58.
124	gb|CDO13981.1|ARO:3003585|Klebsiella	macrolide antibiotic;peptide antibiotic	antibiotic target alteration;antibiotic efflux	Klebsiella mutant PhoP conferring antibiotic resistance to colistin	A mutant phoP activates pmrHFIJKLM expression responsible for L-aminoarabinose synthesis and polymyxin resistance, by way of alteration of negative charge
125	gb|NP_249871.1|ARO:3003896|Pseudomonas	macrolide antibiotic;peptide antibiotic	antibiotic target alteration;antibiotic efflux	Pseudomonas mutant PhoQ conferring resistance to colistin	Mutations in Pseudomonas aeruginosa PhoQ of the two-component PhoPQ regulatory system. Presence of mutation confers resistance to colistin

126	gb|AAV85982.1|ARO:3000535|macB	macrolide antibiotic	antibiotic efflux	macB	MacB is an ATP-binding cassette (ABC) transporter that exports macrolides with 14- or 15- membered lactones. It forms an antibiotic efflux complex with MacA and TolC. macB corresponds to 1 locus in Pseudomonas aeruginosa PAO1 and 1 locus in Pseudomonas aeruginosa LESB58.
127	gb|CCP45025.1|ARO:3003463|Mycobacterium	isoniazid;triclosan	antibiotic target alteration	Mycobacterium tuberculosis kasA mutant conferring resistance to isoniazid	Specific mutations on the Mycobacterium tuberculosis kasA gene resulting in lowered affinity of isoniazid, resulting in resistance
128	gb|NP_415611.1|ARO:3004049|antibiotic	triclosan	antibiotic target alteration	Escherichia coli fabG mutations conferring resistance to triclosan	fabG is a 3-oxoacyl-acyl carrier protein reductase involved in lipid metabolism and fatty acid biosynthesis.The bacterial biocide Triclosan blocks the final reduction step in fatty acid elongation, inhibiting biosynthesis. Point mutations in fabG can confer resistance to Triclosan.

129	gb|AET10444.1|ARO:3004032|tetA(46)	tetracycline antibiotic	antibiotic efflux	tetA(46)	tetA(46) is a subunit of tetAB(46), a heterodimeric ABC transporter, that is required for conferring tetracycline resistance in Streptococcus australis isolated from the oral cavity.
130	gb|ALV80601.1|ARO:3003801|bcr-1	bicyclomycin	antibiotic efflux	bcr-1	Transmembrane protein which expels bicyclomycin from the cell, leading to bicyclomycin resistance. Identified in Pseudomonas aeruginosa strains responsible for outbreaks in Brazil, often appearing with blaSPM-1, another bicyclomycin resistance gene
131	gb|NP_415222.1|ARO:3003841|kdpE	aminoglycoside antibiotic	antibiotic efflux	kdpE	kdpE is a transcriptional activator that is part of the two-component system KdpD/KdpE that is studied for its regulatory role in potassium transport and has been identified as an adaptive regulator involved in the virulence and intracellular survival of pathogenic bacteria. kdpE regulates a range of virulence loci through direct promoter binding.

132	gb|ABV18113.1|ARO:3001329|mdtG	fosfomycin	antibiotic efflux	mdtG	The MdtG protein, also named YceE, appears to be a member of the major facilitator superfamily of transporters, and it has been reported, when overexpressed, to increase fosfomycin and deoxycholate resistances. mdtG is a member of the marA-soxS-rob regulon.
133	gb|YP_009077553.1|ARO:3002680|catB8	phenicol antibiotic	antibiotic inactivation	catB8	catB8 is a plasmid or integron-encoded variant of the cat gene found in Klebsiella pneumoniae, Salmonella typhi and Pseudomonas aeruginosa
134	gb|NP_253464.1|ARO:3003582|basR	peptide antibiotic	antibiotic target alteration	basR	Response regulator for Lipid A modification genes; two-component system involved in polymyxin resistance that senses high extracellular Fe(2+)
135	gb|BAA15934.1|ARO:3000829|baeS	aminoglycoside antibiotic;aminocoumarin antibiotic	antibiotic efflux	baeS	BaeS is a sensor kinase in the BaeSR regulatory system. While it phosphorylates BaeR to increase its activity, BaeS is not necessary for overexpressed BaeR to confer resistance.
136	gb|CAE51745.1|ARO:3000168|tet(D)	tetracycline antibiotic	antibiotic efflux	tet(D)	TetD is a tetracycline efflux pump found exclusively in Gram-negative bacteria.

137	gb|AAC75429.1|ARO:3000833|evgS	macrolide antibiotic;fluoroquinolone antibiotic;penam;tetracycline antibiotic	antibiotic efflux	evgS	EvgS is a sensor protein that phosphorylates the regulatory protein EvgA. evgS corresponds to 1 locus in Pseudomonas aeruginosa PAO1 and 1 locus in Pseudomonas aeruginosa LESB58.
138	gb|NP_461214.1|ARO:3004334|Salmonella	triclosan	antibiotic target alteration	Salmonella enterica gyrA with mutation conferring resistance to triclosan	Point mutations in Salmonella enterica serovar Typhimurium which have been shown to increase the minimum inhibitory concentration of the antibiotic triclosan. It is hypothesized that decreased susceptibility to triclosan in Salmonella gyrA mutants occurs indirectly due to alterations in the stress response pathways.
139	gb|ABA71733.1|ARO:3002972|vanTG	glycopeptide antibiotic	antibiotic target alteration	vanTG	vanTG is a vanT variant found in the vanG gene cluster
140	gb|NP_216963.1|ARO:3004157|Mycobacterium	para-aminosalicylic acid	antibiotic target alteration	Mycobacterium tuberculosis folC with mutation conferring resistance to para-aminosalicylic acid	Point mutations in the dihydrofolate synthetase folC gene shown clinically to confer resistance to p-aminosalicylic acid or other aminosalicylates. Mutations in folC inhibit bioactivation of PAS and thus confer resistance.

141	gb|AHA41499.1|ARO:3002948|vanHO	glycopeptide antibiotic	antibiotic target alteration	vanHO	vanHO is a vanH variant in the vanO gene cluster
142	gb|AAA98298.1|ARO:3002545|AAC(6')-Ia	aminoglycoside antibiotic	antibiotic inactivation	AAC(6')-Ia	AAC(6')-Ia is an aminoglycoside acetyltransferase encoded by plasmids, transposons, integrons in Citrobacter diversus, E. coli, K. pneumoniae, Shigella sonnei, and P. aeruginosa.
143	gb|NP_415611.1|ARO:3004049|antibiotic	triclosan	antibiotic target alteration	Escherichia coli fabG mutations conferring resistance to triclosan	fabG is a 3-oxoacyl-acyl carrier protein reductase involved in lipid metabolism and fatty acid biosynthesis.The bacterial biocide Triclosan blocks the final reduction step in fatty acid elongation, inhibiting biosynthesis. Point mutations in fabG can confer resistance to Triclosan.
144	gb|CDJ72759|ARO:3003899|Escherichia	fosfomycin	antibiotic target alteration	Escherichia coli PtsI with mutation conferring resistance to fosfomycin	PtsI (phosphoenolpyruvate-protein phosphotransferase) is involved in cyclic AMP synthesis, which regulates glpT expression. As a result, mutations to ptsI confer resistance to fosfomycin by affecting the regulation of fosfomycin import.

145	gb|AAV85982.1|ARO:3000535|macB	macrolide antibiotic	antibiotic efflux	macB	MacB is an ATP-binding cassette (ABC) transporter that exports macrolides with 14- or 15- membered lactones. It forms an antibiotic efflux complex with MacA and TolC. macB corresponds to 1 locus in Pseudomonas aeruginosa PAO1 and 1 locus in Pseudomonas aeruginosa LESB58.
146	gb|WP_015892743.1|ARO:3002815|clbB	macrolide antibiotic;lincosamide antibiotic;streptogramin antibiotic;oxazolidinone antibiotic;phenicol antibiotic;pleuromutilin antibiotic	antibiotic target alteration	clbB	clbB is a plasmid-encoded cfr gene found in Bacillus brevis
147	gb|AAR84672.1|ARO:3002925|vanRF	glycopeptide antibiotic	antibiotic target alteration	vanRF	vanRF is a vanR variant found in the vanF gene cluster
148	gb|AAA20117.1|ARO:3000195|tetB(P)	tetracycline antibiotic	antibiotic target protection	tetB(P)	TetB(P) is a tetracycline ribosomal protection protein found on the same operon as tetA(P), a tetracycline efflux protein.

149	gb|WP_000192137.1|ARO:3000838|arlR	fluoroquinolone antibiotic;acridine dye	antibiotic efflux	arlR/hprR	ArlR/HprR is a response regulator that binds to the norA promoter to activate expression. ArlR must first be phosphorylated by ArlS.
150	gb|ALH22601.1|ARO:3000620|adeL	fluoroquinolone antibiotic;tetracycline antibiotic	antibiotic efflux	adeL	AdeL is a regulator of AdeFGH in Acinetobacter baumannii. AdeL mutations are associated with AdeFGH overexpression and multidrug resistance.
151	gb|AAF36802.1|ARO:3002945|vanHF	glycopeptide antibiotic	antibiotic target alteration	vanHF	vanHF is a vanH variant in the vanF gene cluster
152	gb|AAF67494.2|ARO:3002522|novA	aminocoumarin antibiotic	antibiotic efflux	novA	A type III ABC transporter, identified on the novobiocin biosynthetic gene cluster, involved in the transport and resistance of novobiocin.

153	gb|NP_415434.1|ARO:3003950|msbA	nitroimidazole antibiotic	antibiotic efflux	msbA	MsbA is a multidrug resistance transporter homolog from E. coli and belongs to a superfamily of transporters that contain an adenosine triphosphate (ATP) binding cassette (ABC) which is also called a nucleotide-binding domain (NBD). MsbA is a member of the MDR-ABC transporter group by sequence homology. MsbA transports lipid A, a major component of the bacterial outer cell membrane, and is the only bacterial ABC transporter that is essential for cell viability.
154	gb|AAB05626.1|ARO:3002943|vanHB	glycopeptide antibiotic	antibiotic target alteration	vanHB	vanHB is a vanH variant in the vanB gene cluster
155	gb|AJF83452.1|ARO:3003574|LpxC	peptide antibiotic	antibiotic target alteration	LpxC	The LpxC gene is widely known to be involved in the biosynthesis of lipid A in Gram-negative bacteria and mutations to this gene may cause resistance to antimicrobial peptides that target the outer membrane

156	gb|NP_439290|ARO:3004446|Haemophilus	monobactam;carbapenem;cephalosporin;cephamycin;penam	antibiotic target alteration	Haemophilus influenzae PBP3 conferring resistance to beta-lactam antibiotics	PBP3 is a penicillin-binding protein and beta-lactam resistance enzyme encoded by the ftsI gene in Haemophilus influenzae. Mutations in ftsI confer resistance to beta-lactam antibiotics.
157	gb|NP_251182.1|ARO:3000814|MexT	fluoroquinolone antibiotic;diaminopyrimidine antibiotic;phenicol antibiotic	antibiotic efflux	MexT	MexT is a LysR-type transcriptional activator that positively regulates the expression of MexEF-OprN, OprD, and MexS.
158	gb|NP_415611.1|ARO:3004049|antibiotic	triclosan	antibiotic target alteration	Escherichia coli fabG mutations conferring resistance to triclosan	fabG is a 3-oxoacyl-acyl carrier protein reductase involved in lipid metabolism and fatty acid biosynthesis.The bacterial biocide Triclosan blocks the final reduction step in fatty acid elongation, inhibiting biosynthesis. Point mutations in fabG can confer resistance to Triclosan.

159	gb|AAV85982.1|ARO:3000535|macB	macrolide antibiotic	antibiotic efflux	macB	MacB is an ATP-binding cassette (ABC) transporter that exports macrolides with 14- or 15- membered lactones. It forms an antibiotic efflux complex with MacA and TolC. macB corresponds to 1 locus in Pseudomonas aeruginosa PAO1 and 1 locus in Pseudomonas aeruginosa LESB58.
160	gb|AAC60780.1|ARO:3003049|rosB	peptide antibiotic	antibiotic efflux	rosB	rosB is part of an efflux pump/potassium antiporter system (RosAB) in Yersinia that confers resistance to cationic antimicrobial peptides such as polymyxin B.
161	gb|AFK58562.1|ARO:3003078|Enterococcus	peptide antibiotic	antibiotic target alteration	Enterococcus faecium liaR mutant conferring daptomycin resistance	liaR is a response regulator found in the liaFSR signal transduction pathway. Mutations confer daptomycin resistance.
162	gb|AQA08261.1|ARO:3004097|Streptococcus	peptide antibiotic	antibiotic target alteration	Streptococcus mitis CdsA with mutation conferring daptomycin resistance	CdsA is a phosphatidate cytidylyltransferase which plays a role in the production of membrane phosphatidylglycerol and cardiolipin.

163	gb|ALH22601.1|ARO:3000620|adeL	fluoroquinolone antibiotic;tetracycline antibiotic	antibiotic efflux	adeL	AdeL is a regulator of AdeFGH in Acinetobacter baumannii. AdeL mutations are associated with AdeFGH overexpression and multidrug resistance.
164	gb|YP_006374661.1|ARO:3003438|Enterococcus	elfamycin antibiotic	antibiotic target alteration	Enterococcus faecium EF-Tu mutants conferring resistance to GE2270A	Sequence variants of Enterococcus faecium elongation factor Tu that confer resistance to GE2270A
165	gb|AAV85982.1|ARO:3000535|macB	macrolide antibiotic	antibiotic efflux	macB	MacB is an ATP-binding cassette (ABC) transporter that exports macrolides with 14- or 15- membered lactones. It forms an antibiotic efflux complex with MacA and TolC. macB corresponds to 1 locus in Pseudomonas aeruginosa PAO1 and 1 locus in Pseudomonas aeruginosa LESB58.

166	gb|NP_415611.1|ARO:3004049|antibiotic	triclosan	antibiotic target alteration	Escherichia coli fabG mutations conferring resistance to triclosan	fabG is a 3-oxoacyl-acyl carrier protein reductase involved in lipid metabolism and fatty acid biosynthesis.The bacterial biocide Triclosan blocks the final reduction step in fatty acid elongation, inhibiting biosynthesis. Point mutations in fabG can confer resistance to Triclosan.
167	gb|AJF82049.1|ARO:3003573|LpxA	peptide antibiotic	antibiotic target alteration	LpxA	The LpxA gene is widely known to be involved in the biosynthesis of lipid A in Gram-negative bacteria and mutations to this gene may cause resistance to antimicrobial peptides that target the outer membrane
168	gb|AQA08261.1|ARO:3004097|Streptococcus	peptide antibiotic	antibiotic target alteration	Streptococcus mitis CdsA with mutation conferring daptomycin resistance	CdsA is a phosphatidate cytidylyltransferase which plays a role in the production of membrane phosphatidylglycerol and cardiolipin.
169	gb|CAA79966.1|ARO:3003665|NmcR	carbapenem;cephalosporin;cephamycin;penam	antibiotic inactivation	NmcR	NmcR is a homolog of the LysR regulator found in Enterobacter cloacae that contribute to the regulation of NmcA beta-lactamase

170	gb|CDJ72759|ARO:3003899|Escherichia	fosfomycin	antibiotic target alteration	Escherichia coli PtsI with mutation conferring resistance to fosfomycin	PtsI (phosphoenolpyruvate-protein phosphotransferase) is involved in cyclic AMP synthesis, which regulates glpT expression. As a result, mutations to ptsI confer resistance to fosfomycin by affecting the regulation of fosfomycin import.
171	gb|NP_415434.1|ARO:3003950|msbA	nitroimidazole antibiotic	antibiotic efflux	msbA	MsbA is a multidrug resistance transporter homolog from E. coli and belongs to a superfamily of transporters that contain an adenosine triphosphate (ATP) binding cassette (ABC) which is also called a nucleotide-binding domain (NBD). MsbA is a member of the MDR-ABC transporter group by sequence homology. MsbA transports lipid A, a major component of the bacterial outer cell membrane, and is the only bacterial ABC transporter that is essential for cell viability.
172	gb|AAA25550.1|ARO:3003105|dfrA3	diaminopyrimidine antibiotic	antibiotic target replacement	dfrA3	dfrA3 is an integron-encoded dihydrofolate reductase found in Escherichia coli

173	gb|NP_462089.1|ARO:3003939|Salmonella	fluoroquinolone antibiotic	antibiotic target alteration	Salmonella enterica parC conferring resistance to fluoroquinolones	Point mutations in Salmonella parC gene implicated in decreased susceptibility to fluoroquinolone antibiotics, primarily ciprofloxacin and nalidixic acid.
174	gb|WP_000195296.1|ARO:3003316|Escherichia	fluoroquinolone antibiotic	antibiotic target alteration	Escherichia coli parE conferring resistance to fluoroquinolones	Point mutation in Escherichia coli parE resulting in fluoroquinolones resistance
175	gb|ACN32294.1|ARO:3000237|tolC	macrolide antibiotic;fluoroquinolone antibiotic;cephalosporin;glycylcycline;cephamycin;penam;tetracycline antibiotic;aminocoumarin antibiotic;rifamycin antibiotic;phenicol antibiotic;triclosan	antibiotic efflux	TolC	TolC is a protein subunit of many multidrug efflux complexes in Gram negative bacteria. It is an outer membrane efflux protein and is constitutively open. Regulation of efflux activity is often at its periplasmic entrance by other components of the efflux complex.
176	gb|AAR84672.1|ARO:3002925|vanRF	glycopeptide antibiotic	antibiotic target alteration	vanRF	vanRF is a vanR variant found in the vanF gene cluster

177	gb|CDJ73208|ARO:3003890|Escherichia	fosfomycin	antibiotic target alteration	Escherichia coli UhpT with mutation conferring resistance to fosfomycin	Mutations to the active importer UhpT, which is involved with the uptake of many phosphorylated sugars, confer resistance to fosfomycin by reducing import of the drug into the bacteria.
178	gb|CAG39573|ARO:3003735|Staphylococcus	fusidic acid	antibiotic target alteration	Staphylococcus aureus fusA with mutation conferring resistance to fusidic acid	The mutations to this gene are involved in altering the translation elongation factor G (EF-G) in association with the ribosome to prevent fusidic acid from binding EF-G and preventing translation.
179	gb|CDO61516.1|ARO:3003949|efrB	macrolide antibiotic;fluoroquinolone antibiotic;rifamycin antibiotic	antibiotic efflux	efrB	efrB is a part of the EfrAB efflux pump, and both efrA and efrB are necessary to confer multidrug resistance.

180	gb|AAV85982.1|ARO:3000535|macB	macrolide antibiotic	antibiotic efflux	macB	MacB is an ATP-binding cassette (ABC) transporter that exports macrolides with 14- or 15- membered lactones. It forms an antibiotic efflux complex with MacA and TolC. macB corresponds to 1 locus in Pseudomonas aeruginosa PAO1 and 1 locus in Pseudomonas aeruginosa LESB58.
181	gb|NP_415434.1|ARO:3003950|msbA	nitroimidazole antibiotic	antibiotic efflux	msbA	MsbA is a multidrug resistance transporter homolog from E. coli and belongs to a superfamily of transporters that contain an adenosine triphosphate (ATP) binding cassette (ABC) which is also called a nucleotide-binding domain (NBD). MsbA is a member of the MDR-ABC transporter group by sequence homology. MsbA transports lipid A, a major component of the bacterial outer cell membrane, and is the only bacterial ABC transporter that is essential for cell viability.

182	gb|ANK04027.1|ARO:3003838|gadW	macrolide antibiotic;fluoroquinolone antibiotic;penam	antibiotic efflux	gadW	GadW is an AraC-family regulator that promotes mdtEF expression to confer multidrug resistance. GadW inhibits GadX-dependent activation. GadW clearly represses gadX and, in situations where GadX is missing, activates gadA and gadBC.
183	gb|CAE51745.1|ARO:3000168|tet(D)	tetracycline antibiotic	antibiotic efflux	tet(D)	TetD is a tetracycline efflux pump found exclusively in Gram-negative bacteria.
184	gb|AAA99504.1|ARO:3002987|bcrA	peptide antibiotic	antibiotic efflux	bcrA	bcrA is an ABC transporter found in Bacillus licheniformis that confers bacitracin resistance
185	gb|AAC77033.1|ARO:3003381|Escherichia	fluoroquinolone antibiotic;cephalosporin;glycylcycline;penam;tetracycline antibiotic;rifamycin antibiotic;phenicol antibiotic;triclosan	antibiotic target alteration;antibiotic efflux	Escherichia coli soxR with mutation conferring antibiotic resistance	SoxR is a sensory protein that upregulates soxS expression in the presence of redox-cycling drugs. This stress response leads to the expression many multidrug efflux pumps.

186	gb|NP_252894.1|ARO:3000806|mexG	fluoroquinolone antibiotic;tetracycline antibiotic;acridine dye	antibiotic efflux	MexG	MexG is a membrane protein required for MexGHI-OpmD efflux activity.
187	gb|NP_252895.1|ARO:3000807|mexH	fluoroquinolone antibiotic;tetracycline antibiotic;acridine dye	antibiotic efflux	MexH	MexH is the membrane fusion protein of the efflux complex MexGHI-OpmD.
188	gb|NP_252896.1|ARO:3000808|mexI	fluoroquinolone antibiotic;tetracycline antibiotic;acridine dye	antibiotic efflux	MexI	MexI is the inner membrane transporter of the efflux complex MexGHI-OpmD.
189	gb|NP_253677.1|ARO:3004038|Pseudomonas	aminoglycoside antibiotic	antibiotic efflux	Pseudomonas aeruginosa emrE	EmrE is a small multidrug transporter that functions as a homodimer and that couples the efflux of small polyaromatic cations from the cell with the import of protons down an electrochemical gradient. Confers resistance to tetraphenylphosphonium, methyl viologen, gentamicin, kanamycin, and neomycin.

190	gb|ACJ59254.1|ARO:3000768|abeS	macrolide antibiotic;aminocoumarin antibiotic	antibiotic efflux	abeS	AbeS in an efflux pump of the SMR family of transporters found in Acinetobacter baumannii.
191	gb|CAC35723.1|ARO:3003374|Enterobacter	fluoroquinolone antibiotic;cephalosporin;glycylcycline;penam;tetracycline antibiotic;rifamycin antibiotic;phenicol antibiotic;triclosan	antibiotic target alteration;antibiotic efflux	Enterobacter aerogenes acrR with mutation conferring multidrug antibiotic resistance	AcrR is a repressor of the AcrAB-TolC multidrug efflux complex. AcrR mutations result in high level antibiotic resistance.
192	gb|AAV85982.1|ARO:3000535|macB	macrolide antibiotic	antibiotic efflux	macB	MacB is an ATP-binding cassette (ABC) transporter that exports macrolides with 14- or 15- membered lactones. It forms an antibiotic efflux complex with MacA and TolC. macB corresponds to 1 locus in Pseudomonas aeruginosa PAO1 and 1 locus in Pseudomonas aeruginosa LESB58.
193	gb|AAD22403.1|ARO:3002970|vanTC	glycopeptide antibiotic	antibiotic target alteration	vanTC	vanTC is a vanT variant found in the vanC gene cluster

194	gb|YP_039997.1|ARO:3003291|Staphylococcus	peptide antibiotic	antibiotic target alteration	Staphylococcus aureus rpoC conferring resistance to daptomycin	Point mutations that occurs in Staphylococcus aureus rpoC resulting in resistance to daptomycin
195	gb|NP_312937.1|ARO:3003288|Escherichia	peptide antibiotic;rifamycin antibiotic	antibiotic target alteration;antibiotic target replacement	Escherichia coli rpoB mutants conferring resistance to rifampicin	Point mutations that occurs in Escherichia coli rpoB resulting in resistance to rifampicin
196	gb|AAA50993.1|ARO:3003368|Escherichia	elfamycin antibiotic	antibiotic target alteration	Escherichia coli EF-Tu mutants conferring resistance to kirromycin	Sequence variants of Escherichia coli elongation factor Tu that confer resistance to kirromycin
197	gb|CAG39573|ARO:3003735|Staphylococcus	fusidic acid	antibiotic target alteration	Staphylococcus aureus fusA with mutation conferring resistance to fusidic acid	The mutations to this gene are involved in altering the translation elongation factor G (EF-G) in association with the ribosome to prevent fusidic acid from binding EF-G and preventing translation.
198	gb|AAK44936.1|ARO:3003395|Mycobacterium	aminoglycoside antibiotic	antibiotic target alteration	Mycobacterium tuberculosis rpsL mutations conferring resistance to Streptomycin	Ribosomal protein S12 stabilizes the highly conserved

199	gb|AKA86814|ARO:3003746|optrA	oxazolidinone antibiotic	antibiotic target protection	optrA	optrA encodes an ABC-transporter gene conferring resistance to oxazolidinones that was isolated from a plasmid in Enterococcus faecalis and Enterococcus faecium.
200	gb|BAE77933.1|ARO:3000518|CRP	macrolide antibiotic;fluoroquinolone antibiotic;penam	antibiotic efflux	CRP	CRP is a global regulator that represses MdtEF multidrug efflux pump expression.
201	gb|NP_417544.5|ARO:3000024|patA	fluoroquinolone antibiotic	antibiotic efflux	patA	PatA is an ABC transporter of Streptococcus pneumoniae that interacts with PatB to confer fluoroquinolone resistance.
202	gb|AAV85982.1|ARO:3000535|macB	macrolide antibiotic	antibiotic efflux	macB	MacB is an ATP-binding cassette (ABC) transporter that exports macrolides with 14- or 15- membered lactones. It forms an antibiotic efflux complex with MacA and TolC. macB corresponds to 1 locus in Pseudomonas aeruginosa PAO1 and 1 locus in Pseudomonas aeruginosa LESB58.

203	gb|AAV85982.1|ARO:3000535|macB	macrolide antibiotic	antibiotic efflux	macB	MacB is an ATP-binding cassette (ABC) transporter that exports macrolides with 14- or 15- membered lactones. It forms an antibiotic efflux complex with MacA and TolC. macB corresponds to 1 locus in Pseudomonas aeruginosa PAO1 and 1 locus in Pseudomonas aeruginosa LESB58.
204	gb|CAA37477.1|ARO:3002891|otr(A)	tetracycline antibiotic	antibiotic target protection	otr(A)	otr(A) is an oxytetracycline resistance ribosomal protection protein found in Streptomyces rimosus
205	gb|YP_499945.1|ARO:3000839|arlS	fluoroquinolone antibiotic;acridine dye	antibiotic efflux	arlS	ArlS is a protein histidine kinase that phosphorylates ArlR, a promoter for norA expression.
206	gb|WP_011461303.1|ARO:3003728|vanRI	glycopeptide antibiotic	antibiotic target alteration	vanRI	VanRI is the regulatory transcriptional activator in the VanSR regulator within the VanI glycopeptide resistance gene cluster.
207	gb|NP_312864.1|ARO:3000830|cpxA	aminoglycoside antibiotic;aminocoumarin antibiotic	antibiotic efflux	cpxA	CpxA is a membrane-localized sensor kinase that is activated by envelope stress. It starts a kinase cascade that activates CpxR, which promotes efflux complex expression.

208	gb|AAV85982.1|ARO:3000535|macB	macrolide antibiotic	antibiotic efflux	macB	MacB is an ATP-binding cassette (ABC) transporter that exports macrolides with 14- or 15- membered lactones. It forms an antibiotic efflux complex with MacA and TolC. macB corresponds to 1 locus in Pseudomonas aeruginosa PAO1 and 1 locus in Pseudomonas aeruginosa LESB58.
209	gb|APB03219.1|ARO:3003986|TaeA	pleuromutilin antibiotic	antibiotic efflux	TaeA	Pleuromutilin (Tiamulin) ABC efflux pump found in Paenibacillus sp. LC231, a strain of Paenibacillus isolated from Lechuguilla Cave, NM, USA. Confers resistance to pleuromutilin antibiotics. Described by Pawlowski et al. 2016.
210	gb|AET10444.1|ARO:3004032|tetA(46)	tetracycline antibiotic	antibiotic efflux	tetA(46)	tetA(46) is a subunit of tetAB(46), a heterodimeric ABC transporter, that is required for conferring tetracycline resistance in Streptococcus australis isolated from the oral cavity.

211	gb|AAV85982.1|ARO:3000535|macB	macrolide antibiotic	antibiotic efflux	macB	MacB is an ATP-binding cassette (ABC) transporter that exports macrolides with 14- or 15- membered lactones. It forms an antibiotic efflux complex with MacA and TolC. macB corresponds to 1 locus in Pseudomonas aeruginosa PAO1 and 1 locus in Pseudomonas aeruginosa LESB58.
212	gb|BAJ09383.1|ARO:3003046|qacA	fluoroquinolone antibiotic	antibiotic efflux	qacA	qacA is a subunit of the qac multidrug efflux pump
213	gb|NP_415611.1|ARO:3004049|antibiotic	triclosan	antibiotic target alteration	Escherichia coli fabG mutations conferring resistance to triclosan	fabG is a 3-oxoacyl-acyl carrier protein reductase involved in lipid metabolism and fatty acid biosynthesis.The bacterial biocide Triclosan blocks the final reduction step in fatty acid elongation, inhibiting biosynthesis. Point mutations in fabG can confer resistance to Triclosan.
214	gb|AFC91828.1|ARO:3003041|Streptococcus	monobactam;carbapenem;cephalosporin;cephamycin;penam	antibiotic target alteration	Streptococcus pneumoniae PBP1a conferring resistance to amoxicillin	PBP1a is a penicillin-binding protein found in Streptococcus pneumoniae

215	gb|BAA11237.1|ARO:3000254|emrY	tetracycline antibiotic	antibiotic efflux	emrY	emrY is a multidrug transport that moves substrates across the inner membrane of the Gram-negative E. coli. It is a homolog of emrB.
216	gb|CCP46748.1|ARO:3003470|Mycobacterium	aminoglycoside antibiotic	antibiotic target alteration	Mycobacterium tuberculosis gidB mutation conferring resistance to streptomycin	Specific mutations that occurs on Mycobacterium tuberculosis gidB causing it to be streptomycin resistant
217	gb|AAA22081.1|ARO:3004451|Agrobacterium	phenicol antibiotic	antibiotic inactivation	Agrobacterium fabrum chloramphenicol acetyltransferase	A chloramphenicol resistance determinant described in the Gram-negative bacterium Agrobacterium fabrum.
218	gb|CAA79966.1|ARO:3003665|NmcR	carbapenem;cephalosporin;cephamycin;penam	antibiotic inactivation	NmcR	NmcR is a homolog of the LysR regulator found in Enterobacter cloacae that contribute to the regulation of NmcA beta-lactamase
