# Supplementary material for: Unveiling Genomic Islands Hosting Antibiotic Resistance Genes and Virulence Genes in Foodborne Multidrug-Resistant Patho-Genic Proteus vulgaris
Source: Biology (Basel). 2025 Jul 15;14(7):858. doi: 10.3390/biology14070858 (PMC12292103; doi:10.3390/biology14070858)
Supplement: Supplementary file 1 [file biology-14-00858-s001.zip › Table S4-revised.pdf]

Table S4 IS sequence on the P3M genome.

No.	Name	Product	Location (5’-3’)	Length (bp)
1	--	IS3 transposase	12431-12511	81
2	TnpA	IS200/IS605 family transposase	79637-80063	427
3	TnpB	IS200/IS605 family element transposase accessory protein TnpB	80120-81285	1166
4	--	Transposase	339053-339247	195
5	TnpA	IS200/IS605 family transposase	466409-466807	399
6	TnpB	IS200/IS605 family element transposase accessory protein TnpB	467416-468030	615
7	TnpB	IS200/IS605 family element transposase accessory protein TnpB	574576-575744	1169
8	TnpA	IS200/IS605 family transposase	575801-576226	426
9	TnpA	IS200/IS605 family transposase	908812-909237	426
10	TnpB	IS200/IS605 family element transposase accessory protein TnpB	909294-910463	1170
11	--	IS256 family transposase	956170-957379	1210
12	TnpA	IS200/IS605 family transposase	1121669-1122094	426
13	TnpB	IS200/IS605 family element transposase accessory protein TnpB	1122151-1123318	1168
14	TnpA	IS200/IS605 family transposase	1549150-1549575	426

15	TnpB	IS200/IS605 family element transposase accessory protein TnpB	1549631-1550800	1170
16	TnpA	IS200/IS605 family transposase	1609223-1609618	396
17	TnpB	IS200/IS605 family element transposase accessory protein TnpB	1609703-1610869	1167
18	TnpA	IS200/IS605 family transposase	1637581-1638006	426
19	TnpB	IS200/IS605 family element transposase accessory protein TnpB	1638063-1639229	1167
20	TnpB	IS200/IS605 family element transposase accessory protein TnpB	1676470-1677639	1170
21	TnpA	IS200/IS605 family transposase	1677696-1678121	426
22	TnpB	IS200/IS605 family element transposase accessory protein TnpB	1836639-1837806	1168
23	TnpA	IS200/IS605 family transposase	1837862-1838285	424
24	TnpA	IS200/IS605 family transposase	2233940-2234404	467
25	--	Transposase	2305426-2305598	172
26	TnpA	IS200/IS605 family transposase	2625396-2625821	426
27	TnpB	IS200/IS605 family element transposase accessory protein TnpB	2625878-2627046	1169
28	TnpA	IS200/IS605 family transposase	2694588-2694983	396
29	TnpB	IS200/IS605 family element transposase accessory protein TnpB	2695069-2696235	1167
30	TnpB	IS200/IS605 family element transposase accessory protein TnpB	2821424-2822592	1169
31	TnpA	IS200/IS605 family transposase	2822649-2823074	426

32	TnpA	IS200/IS605 family transposase	3259349-3259774	426
33	TnpB	IS200/IS605 family element transposase accessory protein TnpB	3259831-3260998	1168
34	TnpA	IS200/IS605 family transposase	3306858-3307256	399
35	TnpB	IS200/IS605 family element transposase accessory protein TnpB	3307313-3308482	1170
36	TnpA	IS200/IS605 family transposase	3338458-3338883	426
37	TnpB	IS200/IS605 family element transposase accessory protein TnpB	3339286-3340023	738
38	TnpA	IS200/IS605 family transposase	3354532-3354929	690
39	TnpB	IS200/IS605 family element transposase accessory protein TnpB	3355240-3356151	912
40	--	Putative transposase	3564237-3565181	945
41	TnpA	IS200/IS605 family transposase	3814592-3815016	425
42	--	Transposase	3815073-3815723	651
