# Supplementary material for: Unveiling Genomic Islands Hosting Antibiotic Resistance Genes and Virulence Genes in Foodborne Multidrug-Resistant Patho-Genic Proteus vulgaris
Source: Biology (Basel). 2025 Jul 15;14(7):858. doi: 10.3390/biology14070858 (PMC12292103; doi:10.3390/biology14070858)
Supplement: Supplementary file 1 [file biology-14-00858-s001.zip › Table S5-revised.pdf]

Table S5 Virulence genes on the P3M genome. VFDB predictions with ≥70% identity, ≥50% coverage thresholds.

No.	Nr_ID	Nr_Description	Gene_Name	COG_Description
1	WP_109406818.1	antitoxin [Proteus vulgaris]	mvpT, vapB	Virulence-associated protein and related proteins
2	WP_109407568.1	murein biosynthesis integral membrane protein MurJ [Proteus genomosp. 4]	murJ, mviN	Uncharacterized membrane protein, putative virulence factor
3	EEG83014.1	integral membrane protein MviN [Proteus penneri ATCC 35198]	mviN	Uncharacterized membrane protein, putative virulence factor
4	WP_102949457.1	virulence protein [Proteus mirabilis]	--	--
5	WP_109395958.1	virulence factor SrfB [Proteus sp. TJ1636]	srfB	Uncharacterized protein conserved in bacteria, putative virulence factor
6	WP_109395958.1	virulence factor SrfB [Proteus sp. TJ1636]	srfB	Uncharacterized protein conserved in bacteria, putative virulence factor
7	WP_109406748.1	type III secretion system effector [Proteus vulgaris]	srfC	Uncharacterized protein conserved in bacteria, putative virulence factor
8	CRL65324.1	putative DNA-binding transcriptional regulator [Proteus vulgaris]	hipA	Toxin module HipA, protein kinase of phosphatidylinositol 3/4-kinase superfamily
9	WP_072063734.1	RTX toxin [Proteus vulgaris]		RTX toxins and related Ca^{2+}-binding proteins
10	WP_109399333.1	hypothetical protein [Proteus sp. TJ1640]		RTX toxins and related Ca^{2+}-binding proteins
11	WP_109397008.1	MULTISPECIES: type I secretion C-terminal target domain-containing protein [Proteus]	prtC	RTX toxins and related Ca^{2+}-binding proteins
12	WP_109850449.1	metalloprotease [Proteus sp. CA142267]		RTX toxins and related Ca^{2+}-binding proteins

13	WP_109397236.1	MULTISPECIES: hypothetical protein [Proteus]	hicB	Predicted nuclease of the RNAse H fold, HicB family
14	WP_049582328.1	type II toxin-antitoxin system HicA family toxin [Photorhabdus luminescens]		
15	WP_109396049.1	MULTISPECIES: GhoT/OrtT family toxin [Proteus]		
16	WP_072063170.1	toxin [Proteus vulgaris]		
17	WP_023581692.1	MULTISPECIES: colicin V production protein [Enterobacterales]	cvpA	Uncharacterized membrane protein, required for colicin V production
18	WP_006533588.1	MULTISPECIES: type II toxin-antitoxin system RatA family toxin [Proteus]		Oligoketide cyclase/lipid transport protein
19	EEG85338.1	TPR repeat region [Proteus penneri ATCC 35198]	cptB	Uncharacterized conserved protein
20	WP_023583757.1	MULTISPECIES: Hcp family type VI secretion system effector [Enterobacterales]	hcp	Type VI protein secretion system component Hcp (secreted cytotoxin)
21	SPY37344.1	Ser-Thr-rich glycosyl-phosphatidyl-inositol-anchored membrane family [Proteus mirabilis]		RTX toxins and related Ca^{2+}-binding proteins
22	WP_109407025.1	toxin [Proteus vulgaris]	tccC	Rhs family protein
23	WP_115065638.1	RHS repeat protein [Proteus penneri]	tccC	Rhs family protein
24	WP_036914126.1	MULTISPECIES: toxin RelE [Proteus]	relE	Uncharacterized protein conserved in bacteria
25	EST57186.1	hypothetical protein K151_2989 [Proteus hauseri ZMd44]	--	--

26	WP_066746378.1	flagellin FliC [Cosenzaea myxofaciens]	fliC, flgL	Flagellin and related hook-associated proteins
27	WP_036912728.1	MULTISPECIES: RNA polymerase sigma factor FliA [Enterobacterales]	fliA	DNA-directed RNA polymerase specialized sigma subunit
28	WP_075673009.1	MULTISPECIES: flagellar regulatory protein FliZ [Proteus]	fliZ	Regulator of sigma S factor FliZ
29	WP_109396129.1	MULTISPECIES: flagellar protein regulator [Proteus]		Predicted outer membrane lipoprotein
30	CRL60252.1	Flagellin 2 [Proteus vulgaris]	flgL	Flagellin and related hook-associated proteins
31	WP_115370765.1	FliC/FljB family flagellin [Proteus vulgaris]	fliC, flgL	Flagellin and related hook-associated proteins
32	WP_109396454.1	MULTISPECIES: flagellar filament capping protein FliD [Proteus]	fliD	Flagellar capping protein
33	WP_109396454.1	MULTISPECIES: flagellar filament capping protein FliD [Proteus]	fliD	Flagellar capping protein
34	WP_109396455.1	MULTISPECIES: flagella export chaperone FliS [Proteus]	fliS	Flagellin-specific chaperone FliS
35	WP_109396456.1	MULTISPECIES: flagella biosynthesis regulatory protein FliT [Proteus]	fliT	Flagellar protein FliT
36	WP_109396460.1	MULTISPECIES: flagellar hook-basal body complex protein FliE [Proteus]	fliE	Flagellar hook-basal body protein
37	WP_109396461.1	MULTISPECIES: flagellar basal body M-ring protein FliF [Proteus]	fliF	Flagellar basal body M-ring protein
38	WP_086108275.1	flagellar motor switch protein FliG [Xenorhabdus vietnamensis]	fliG	Flagellar motor switch protein
39	KLU18997.1	flagellar assembly protein FliH [Proteus mirabilis]	fliH	Flagellar biosynthesis/type III secretory pathway protein

40	WP_036969825.1	flagellum-specific ATP synthase FliL [Proteus mirabilis]	fliI	Flagellar biosynthesis/type III secretory pathway ATPase
41	WP_006533149.1	MULTISPECIES: flagella biosynthesis chaperone FliJ [Enterobacterales]	fliJ	Flagellar biosynthesis chaperone
42	WP_109847291.1	flagellar hook-length control protein FliK [Proteus penneri]	fliK	Flagellar hook-length control protein
43	WP_006533151.1	MULTISPECIES: flagellar basal body-associated protein FliL [Proteus]	fliL	Flagellar basal body-associated protein
44	WP_038192918.1	flagellar motor switch protein FliM [Xenorhabdus bovienii]	fliM	Flagellar motor switch protein
45	WP_006533153.1	MULTISPECIES: flagellar motor switch protein FliN [Enterobacterales]	fliN	Flagellar motor switch/type III secretory pathway protein
46	WP_109396466.1	MULTISPECIES: flagellar biosynthetic protein FliO [Proteus]	fliO	Flagellar biogenesis protein
47	WP_041381992.1	flagellar biosynthetic protein FliP [Photorhabdus asymbiotica]	fliP	Flagellar biosynthesis pathway, component FliP
48	WP_006533156.1	MULTISPECIES: flagellar biosynthetic protein FliQ [Proteus]	fliQ	Flagellar biosynthesis pathway, component FliQ
49	WP_072063103.1	MULTISPECIES: flagellar type III secretion system protein FliR [Proteus]	fliR	Flagellar biosynthesis pathway, component FliR
50	WP_083954530.1	fimbrial protein [Cosenzaea myxofaciens]	fimA	P pilus assembly protein, pilin FimA
51	WP_036895539.1	type 1 fimbrial protein [Proteus mirabilis]	fimA	P pilus assembly protein, pilin FimA
52	WP_066746949.1	fimbrial protein [Cosenzaea myxofaciens]	--	--

53	WP_115065337.1	fimbrial protein [Proteus penneri]	fimC	P pilus assembly protein, chaperone PapD
54	WP_008912509.1	fimbrial outer membrane usher protein [Providencia burhodogranariea]	fimD	P pilus assembly protein, porin PapC
55	WP_083954529.1	adhesin [Cosenzaea myxofaciens]	--	--
56	WP_109396470.1	MULTISPECIES: flagellar hook-filament junction protein FlgL [Proteus]	flgL	Flagellin and related hook-associated proteins
57	WP_109406692.1	flagellar hook-associated protein FlgK [Proteus vulgaris]	flgK	Flagellar hook-associated protein
58	WP_109391458.1	flagellar hook-associated protein FlgK [Proteus sp. FJ2001126-3]	flgK	Flagellar hook-associated protein
59	WP_109396472.1	MULTISPECIES: flagellar assembly peptidoglycan hydrolase FlgJ [Proteus]	flgJ	Muramidase (flagellum-specific)
60	WP_066746487.1	flagellar basal body P-ring protein FlgI [Cosenzaea myxofaciens]	flgI	Flagellar basal-body P-ring protein
61	WP_109396473.1	MULTISPECIES: flagellar basal body L-ring protein FlgH [Proteus]	flgH	Flagellar basal body L-ring protein
62	WP_066746495.1	flagellar basal-body rod protein FlgG [Cosenzaea myxofaciens]	flgG	Flagellar basal body rod protein
63	WP_104731695.1	flagellar basal body rod protein FlgF [Proteus mirabilis]	flgF	Flagellar basal body rod protein
64	WP_109396475.1	flagellar hook protein FlgE [Proteus sp. TJ1636]	flgE	Flagellar hook protein FlgE
65	WP_109396476.1	MULTISPECIES: flagellar hook assembly protein FlgD [Proteus]	flgD	Flagellar hook capping protein
66	WP_006533173.1	MULTISPECIES: flagellar basal body rod protein FlgC [Proteus]	flgC	Flagellar basal body rod protein

67	WP_006533174.1	MULTISPECIES: flagellar basal body rod protein FlgB [Proteus]	flgB	Flagellar basal body protein
68	WP_109396478.1	MULTISPECIES: flagellar basal body P-ring formation protein FlgA [Proteus]	flgA	Flagellar basal body P-ring biosynthesis protein
69	WP_109396479.1	MULTISPECIES: anti-sigma-28 factor FlgM [Proteus]	flgM	Negative regulator of flagellin synthesis
70	WP_109396495.1	MULTISPECIES: flagellar biosynthesis protein FlgN [Proteus]	flgN	Flagellar biosynthesis/type III secretory pathway chaperone
71	WP_060555543.1	flagellar biosynthesis protein FlhA [Proteus mirabilis]	flhA	Flagellar biosynthesis pathway, component FlhA
72	WP_109396483.1	flagellar type III secretion system protein FlhB [Proteus sp. TJ1636]	flhB	Flagellar biosynthesis pathway, component FlhB
73	WP_109396488.1	MULTISPECIES: motility protein MotB [Proteus]	motB	Flagellar motor protein
74	WP_115827264.1	flagellar motor stator protein MotA [Xenorhabdus cabanillasii]	motA	Flagellar motor component
75	WP_006533207.1	MULTISPECIES: flagellar transcriptional regulator FlhC [Enterobacterales]	flhC	Flagellar transcriptional activator FlhC
76	EEG85841.1	flagellar transcriptional activator (FlhD) [Proteus penneri ATCC 35198]	flhD	Flagellar transcriptional activator FlhD
77	WP_023583012.1	EscU/YscU/HrcU family type III secretion system export apparatus switch protein [Proteus hauseri]	flhB	Flagellar biosynthesis pathway, component FlhB
78	WP_109398299.1	MULTISPECIES: hypothetical protein [Proteus]	fliN	Flagellar motor switch/type III secretory pathway protein

79	WP_072064444.1	FliI/YscN family ATPase [Proteus vulgaris]	fliI	Flagellar biosynthesis/type III secretory pathway ATPase
80	WP_109398304.1	MULTISPECIES: EscV/YscV/HrcV family type III secretion system export apparatus protein [Proteus]	escV	Type III secretory pathway, component EscV
81	WP_109398535.1	MULTISPECIES: flagellar protein [Proteus]	--	--
82	WP_109396996.1	filamentous hemagglutinin N-terminal domain-containing protein, partial [Proteus sp. TJ1636]	fhaB	Large exoproteins involved in heme utilization or adhesion
83	WP_086163708.1	hypothetical protein, partial [Escherichia coli]	fhaB	Filamentous hemagglutinin
84	WP_066749552.1	filamentous hemagglutinin [Cosenzaea myxofaciens]	--	--
85	WP_100159466.1	filamentous hemagglutinin N-terminal domain-containing protein [Proteus columbae]	fhaB	Large exoproteins involved in heme utilization or adhesion
86	WP_072062805.1	filamentous hemagglutinin N-terminal domain-containing protein [Proteus vulgaris]	--	--
87	WP_109406448.1	filamentous hemagglutinin N-terminal domain-containing protein [Proteus vulgaris]	fhaB	Large exoproteins involved in heme utilization or adhesion
88	WP_096863359.1	filamentous hemagglutinin N-terminal domain-containing protein [Providencia rettgeri]	--	--

89	WP_109392277.1	filamentous hemagglutinin N-terminal domain-containing protein [Proteus sp. FJ2001126-3]	--	--
90	WP_109397919.1	filamentous hemagglutinin N-terminal domain-containing protein [Proteus sp. TJ1636]	--	--
91	WP_109406412.1	filamentous hemagglutinin N-terminal domain-containing protein [Proteus vulgaris]	--	--
92	WP_072063116.1	filamentous hemagglutinin N-terminal domain-containing protein [Proteus vulgaris]	--	--
93	WP_109396481.1	filamentous hemagglutinin N-terminal domain-containing protein [Proteus sp. TJ1636]	--	--
94	WP_109406697.1	filamentous hemagglutinin N-terminal domain-containing protein [Proteus vulgaris]	fhaB	Large exoproteins involved in heme utilization or adhesion
95	WP_072069259.1	filamentous hemagglutinin N-terminal domain-containing protein [Proteus vulgaris]	shlA, hhdA, hpmA	Hemolysin
96	WP_109397831.1	MULTISPECIES: cell filamentation protein Fic [Proteus]	fic	Protein involved in cell division
97	WP_109396016.1	MULTISPECIES: cell filamentation protein Fic [Proteus]	fic	--
98	WP_109406722.1	filamentous hemagglutinin N-terminal	--	--

		domain-containing protein [Proteus vulgaris]		
99	CRL65084.1	Filamentous hemagglutinin [Proteus vulgaris]	--	Large exoproteins involved in heme utilization or adhesion
100	WP_023581204.1	ShlB/FhaC/HecB family hemolysin secretion/activation protein [Proteus hauseri]	fhaC	Hemolysin activation/secretion protein
101	WP_109397310.1	MULTISPECIES: transcriptional regulator SlyA [Proteus]	slyA, marR	Transcriptional regulators
102	WP_109392276.1	ShlB/FhaC/HecB family hemolysin secretion/activation protein [Proteus sp. FJ2001126-3]	fhaC	Hemolysin activation/secretion protein
103	WP_109392871.1	hemolysin activation protein [Proteus genomosp. 5]	shlB, hhdB, hpmB	Hemolysin activation/secretion protein
104	WP_109398221.1	YtjB family periplasmic protein [Proteus sp. TJ1636]	ahpA	Uncharacterized membrane protein affecting hemolysin expression
105	KLU18120.1	two-partner secretion system accessory protein [Proteus mirabilis]	fhaC	Hemolysin activation/secretion protein
106	WP_109848041.1	ShlB/FhaC/HecB family hemolysin secretion/activation protein [Proteus penneri]	fhaC	Hemolysin activation/secretion protein
107	WP_036844098.1	MULTISPECIES: HlyC/CorC family transporter [Photorhabdus]	tlyC	Hemolysins and related proteins containing CBS domains
108	WP_006535062.1	MULTISPECIES: hemolysin expression modulator Hha [Enterobacterales]	hha	haemolysin expression modulating protein

109	WP_081045300.1	ShlB/FhaC/HecB family hemolysin secretion/activation protein [Proteus mirabilis]	fhaC	Hemolysin activation/secretion protein
110	WP_006534899.1	MULTISPECIES: type 1 fimbrial protein [Proteus]	fimA	P pilus assembly protein, pilin FimA
111	WP_109397032.1	MULTISPECIES: type 1 fimbrial protein [Proteus]	fimA	P pilus assembly protein, pilin FimA
112	WP_109397029.1	MULTISPECIES: fimbrial protein [Proteus]	fimA	P pilus assembly protein, pilin FimA
113	WP_109397027.1	MULTISPECIES: type 1 fimbrial protein [Proteus]	fimA	P pilus assembly protein, pilin FimA
114	WP_109373867.1	MULTISPECIES: type 1 fimbrial protein [Proteus]	fimA	P pilus assembly protein, pilin FimA
115	WP_109397023.1	MULTISPECIES: type 1 fimbrial protein [Proteus]	fimA	P pilus assembly protein, pilin FimA
116	WP_109397020.1	fimbrial protein [Proteus sp. TJ1636]	fimA	P pilus assembly protein, pilin FimA
117	WP_109397018.1	MULTISPECIES: type 1 fimbrial protein [Proteus]	fimA	P pilus assembly protein, pilin FimA
118	WP_109395717.1	MULTISPECIES: type 1 fimbrial protein [Proteus]	fimA	P pilus assembly protein, pilin FimA
119	WP_006533114.1	fimbrial biogenesis outer membrane usher protein [Proteus penneri]	fimD	P pilus assembly protein, porin PapC
120	WP_109406716.1	fimbrial protein [Proteus vulgaris]	--	--
121	WP_109407632.1	type-1 fimbrial protein subunit A [Proteus genomosp. 4]	fimA	P pilus assembly protein, pilin FimA
122	WP_109407631.1	fimbrial protein [Proteus genomosp. 4]	fimA	P pilus assembly protein, pilin FimA

123	WP_109397653.1	MULTISPECIES: fimbrial protein FimD [Proteus]	fimD, fimC, mrkC, htrE, cssD	P pilus assembly protein, porin PapC
124	WP_109407627.1	type 1 fimbrial protein [Proteus genomosp. 4]	fimA	P pilus assembly protein, pilin FimA
125	CRL62865.1	putative fimbrial protein [Proteus vulgaris]	fimA	P pilus assembly protein, pilin FimA
126	WP_098944071.1	fimbrial biogenesis outer membrane usher protein [Proteus vulgaris]	fimD	P pilus assembly protein, porin PapC
127	WP_072064059.1	fimbrial protein [Proteus vulgaris]	fimA	P pilus assembly protein, pilin FimA
128	WP_109397549.1	MULTISPECIES: fimbrial protein [Proteus]	--	--
129	WP_109847406.1	fimbrial biogenesis outer membrane usher protein [Proteus penneri]	fimD	P pilus assembly protein, porin PapC
130	WP_109397546.1	MULTISPECIES: type 1 fimbrial protein [Proteus]	fimA	P pilus assembly protein, pilin FimA
131	WP_036895536.1	type 1 fimbrial protein [Proteus mirabilis]	fimA	P pilus assembly protein, pilin FimA
132	WP_070926105.1	type 1 fimbrial protein [Providencia stuartii]	fimA	P pilus assembly protein, pilin FimA
133	WP_109396373.1	MULTISPECIES: type 1 fimbrial protein [Proteus]	fimA	P pilus assembly protein, pilin FimA
134	WP_109396370.1	MULTISPECIES: fimbrial protein [Proteus]	fimA	P pilus assembly protein, pilin FimA
135	WP_109396369.1	MULTISPECIES: type 1 fimbrial protein [Proteus]	fimA	P pilus assembly protein, pilin FimA
136	WP_109397823.1	fimbrial protein [Proteus sp. TJ1636]	fimA	P pilus assembly protein, pilin FimA

137	WP_109397824.1	type 1 fimbrial protein [Proteus sp. TJ1636]	fimA	P pilus assembly protein, pilin FimA
138	WP_109397825.1	MULTISPECIES: type 1 fimbrial protein [Proteus]	fimA	P pilus assembly protein, pilin FimA
139	WP_036934766.1	fimbrial biogenesis outer membrane usher protein [Proteus vulgaris]	fimD	P pilus assembly protein, porin PapC
140	WP_006534062.1	MULTISPECIES: ferrous iron transporter B [Proteus]	fimA	P pilus assembly protein, pilin FimA
141	WP_109396241.1	MULTISPECIES: type 1 fimbrial protein [Proteus]	fimA	P pilus assembly protein, pilin FimA
142	WP_109396242.1	MULTISPECIES: type 1 fimbrial protein [Proteus]	fimA	P pilus assembly protein, pilin FimA
143	WP_109396243.1	MULTISPECIES: outer membrane usher protein [Proteus]	fimD	P pilus assembly protein, porin PapC
144	WP_069369660.1	MULTISPECIES: hypothetical protein [Shigella]	fimC	P pilus assembly protein, chaperone PapD
145	WP_109396245.1	MULTISPECIES: type 1 fimbrial protein [Proteus]	fimA	P pilus assembly protein, pilin FimA
146	WP_069369662.1	MULTISPECIES: type 1 fimbrial protein [Shigella]	fimA	P pilus assembly protein, pilin FimA
147	WP_069369663.1	MULTISPECIES: hypothetical protein [Shigella]	--	cell adhesion
148	WP_109396269.1	MULTISPECIES: type 1 fimbrial protein [Proteus]	fimA	P pilus assembly protein, pilin FimA
149	WP_109396270.1	MULTISPECIES: fimbrial protein [Proteus]	fimA	P pilus assembly protein, pilin FimA

150	WP_109396273.1	fimbriae usher protein StfC [Proteus sp. TJ1636]	fimD	P pilus assembly protein, porin PapC
151	WP_109398332.1	MULTISPECIES: fimbrial protein [Proteus]	fimA	P pilus assembly protein, pilin FimA
152	KLU18244.1	type 1 fimbrial chaperone protein [Proteus mirabilis]	fimC	P pilus assembly protein, chaperone PapD
153	WP_109848144.1	fimbrial protein FimD [Proteus penneri]	fimD	P pilus assembly protein, porin PapC
154	WP_109398335.1	type 1 fimbrial protein [Proteus sp. TJ1636]	fimA	P pilus assembly protein, pilin FimA
155	WP_109398336.1	MULTISPECIES: type 1 fimbrial protein [Proteus]	fimA	P pilus assembly protein, pilin FimA
156	WP_109397759.1	MULTISPECIES: fimbrial assembly protein [Proteus]	--	--
